# Complex Signaling Networks Underlying Blue-Light-Mediated Floral Transition in Plants

**DOI:** 10.3390/plants14101533

**Published:** 2025-05-20

**Authors:** Yun Kong, Youbin Zheng

**Affiliations:** School of Environmental Science, University of Guelph, 50 Stone Road East, Guelph, ON N1G 2W1, Canada; yunkong@uoguelph.ca

**Keywords:** flower induction, photoreceptors, signal pathways, physiological mechanisms, future directions

## Abstract

Blue light (BL) is important in regulating floral transition. In a controlled environment production system, BL can be manipulated easily and precisely in aspects like peak wavelength, intensity, duration, and co-action with other wavelengths. However, the results of previous studies about BL-mediated floral transition are inconsistent, which implies that an in-depth critical examination of the relevant physiological mechanisms is necessary. This review consolidates the recent findings on the role of BL in mediating floral transition not only in model plants, such as *Arabidopsis thaliana*, but also in crops, especially horticultural crops. The photoreceptors, floral integrator proteins, signal pathways, and key network components involved in BL-mediated floral transition are critically reviewed. This review provides possible explanations for the contrasting results of previous studies on BL-mediated flowering; it provides valuable information to explain and develop BL manipulation strategies for mediating flowering, especially in horticultural plants. The review also identifies the knowledge gaps and outlines future directions for research in related fields.

## 1. Introduction

Floral transition is a crucial developmental phase that determines the reproductive success of plants and significantly impacts the timing of harvest and marketability in horticultural crops [1]. This transition involves two core features: the induction of floral signals in leaves and their subsequent transport to the shoot apical meristem (SAM), where vegetative growth shifts to reproductive development [2]. For successful flowering, the SAM must finish a transition from an incompetent to a competent state for the formation of floral meristems.

Light spectral quality is one of the important environmental cues regulating floral transition, acting through internal light perception systems [3]. While the role of red and far-red (FR) light has been well-established [4], blue light (BL; 400–500 nm) seems to be a versatile but crucial regulator of flowering [5]. For example, we found that even under 24 h modest-intensity lighting, pure BL promoted flowering of short-day (SD) plants in marigold (*Tagetes erecta*) compared with red light; however, impure BL containing a low level of red light failed to induce flowering, and adding a low level of FR light to the impure BL restored the flowering-promoting effect [6,7]. It appears that BL influences flowering not only through photoperiodism but also by interacting with other light wavelength(s) and intensity [8]. Generally, BL-mediated flowering involves the light signal’s perception by its photoreceptors, which initiate downstream signaling cascades influencing the expression of key flowering genes, such as *CONSTANS (CO)* and *FLOWERING LOCUS T (FT)*, to control floral transition [9,10].

In recent years, advances in light-emitting diode (LED) technology have enabled precise manipulation of light spectral quality, particularly BL, in controlled environment agriculture (CEA). Blue LEDs alone, or their combination with other wavelengths, are increasingly used for night interruption, day extension, supplemental, and sole-source lighting strategies to regulate flowering in various crops [8]. Compared to FRLEDs, blue LEDs are more cost-effective and widely accessible, making them a practical choice for commercial applications [11]. In *Arabidopsis thaliana* (hereafter *Arabidopsis*), BL has been shown to effectively promote flowering via stabilization of the CO protein under long-day (LD) conditions [9,12]. However, conflicting results have been reported across plant species and even within the same species [8]. For example, in LD plants, while LD treatment with BL promoted flowering in petunia (*Petunia × hybrida*), like *Arabidopsis* [13], it had negligible effects in *Gypsophila paniculata* [14]. In the SD plant chrysanthemum (*Chrysanthemum morifolium*), LD treatment with BL failed to inhibit flowering in growth chambers across a wide range of light intensities [15,16,17,18] but suppressed flowering in greenhouse conditions only when the light intensity exceeded a threshold [19,20]. These variable BL-mediated flowering responses (dependent on plant species, environmental conditions, and light intensities) suggest the involvement of a complex underlying molecular signaling mechanism.

Despite growing interest, a comprehensive understanding of the molecular signaling mechanism involved in BL-mediated floral regulation remains limited, especially in economically important horticultural crops. Shibuya [9] has proposed two signaling pathways for BL-promoted flowering in terms of photoreceptors: FKF1 and CRY2. However, light can regulate plant flowering through at least three pathways, photoperiod, shade, and light quantity pathways [21], and BL signals can be precepted by multiple receptors other than FKF1 and CRY2 [5]. This suggests that more than two pathways can be involved in BL-mediated flowering. Also, most mechanistic insights have been derived from model species under idealized conditions, which may not translate directly to crop production systems. Moreover, even in the model plant, *Arabidopsis*, there is insufficient clarity on how the BL signaling network specifically regulates flower transition, despite documentation on light regulation.

This review aims to consolidate current knowledge on the mechanisms underlying light-mediated flowering, with a focus on BL, in both model species and important crops (e.g., horticultural plants). We identify and discuss key photoreceptors, signaling pathways, and gene expression networks involved in this process. Additionally, we try to use the knowledge on the underlying mechanisms to explain the previous conflicting findings on BL-mediated flowering. Finally, we highlight existing knowledge gaps and propose future research directions to improve the application of BL for flowering control in CEA.

## 2. Photoreceptors and Photosynthetic Pigments

**Figure 1:**
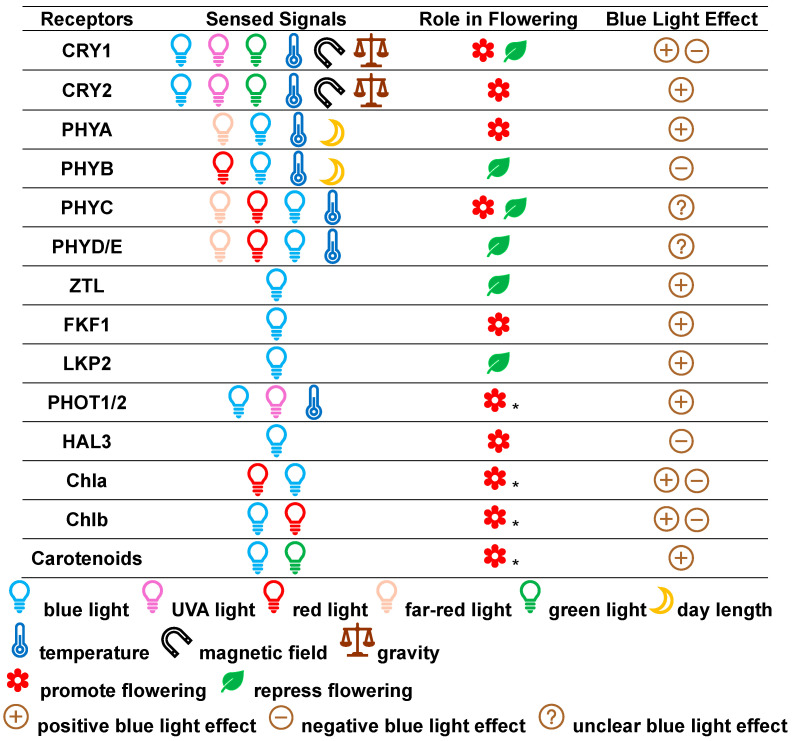
An illustration of the functions of photoreceptors and photosynthetic pigments involved in blue-light-mediated flowering in terms of their sensed signals, role in flowering, and response to blue light. CRY1/2 = cryptochrome1/2; PHYA/B/C/D/E = phytochrome A/B/C/D/E; ZTL = ZEITLUPE; FKF1 = FLAVIN-BINDING, KELCHREPEAT, F-BOX; LKP2 = LOV KELCH PROTEIN2; PHOT1/2 = phototropin1/2; HAL3 = Halotolerance protein; and Chl = Chlorophyll. If both flowering promotion and repression are shown together, this indicates that the role of this receptor depends on environmental conditions or plant genotypes. If both the positive and negative effects of blue light are shown together, this indicates that the blue light effect may vary with light intensity or background light. * indicates that there is only indirect evidence.

BL serves as not only an environmental signal to mediate plant flowering through photoreceptors but also an energy source to drive photosynthesis and produce sugar to affect plant flowering through photosynthetic pigments [22]. These photoreceptors and photosynthetic pigments can sense blue light, together with other signals, to mediate plant flowering (Figure 1).

### 2.1. Photoreceptors

BL can be perceived by plants through various light-responsive proteins (termed photoreceptors), such as cryptochromes (CRYs), phytochromes (PHYs), phototropins (PHOTs), and ZEITLUPE (ZTL) family proteins [5,23]. The activation of the BL photoreceptors is sensitive not only to the wavelength but also to the fluence rate (intensity) and photoperiod [24,25]. These photoreceptors are like a series of switches and dimmers, each modulating the activity of a network that controls a specific pathway to flowering [25,26]. Also, the BL receptors interact in synergistic, antagonistic, and redundant ways with each other [24,26].

#### 2.1.1. Cryptochromes (CRYs)

CRYs are primarily photoreceptors of BL and UVA light as well as greenlight, but they can also coordinate or modulate the perception of other environmental signals, such as temperature, magnetic field, and gravity [5,27,28,29].

Like *Arabidopsis*, almost all higher plants studied have two CRY members, CRY1 and CRY2, despite having more than two genes [28,30]. For example, tomato (*Solanum lycopersicum*) and barley (*Hordeum vulgare*) each have at least three CRY genes, *CRY1a*, *CRY1b*, and *CRY2* [31,32]. There is now evidence for CRY3 in *Arabidopsis*, but its function is presently unknown [33,34]. CRY1 is stably expressed in the BL and biologically activated at a higher light intensity, but CRY2 is degraded upon exposure to BL, and it has a more specialized role under conditions of limiting BL intensity [34,35]. In *Arabidopsis*, CRY1 functions in both the nucleus and the cytoplasm, whereas CRY2 seems to be an exclusively nuclear protein that completes its post-translational cycle in the nucleus [34]. In onion (*Allium cepa*), AcCRY1 demonstrates cytoplasmic localization under BL conditions, while it localizes in the nucleus during darkness, indicating a strong dependence on BL for its subcellular distribution [36]. In addition, CRY2 forms photobodies in the nucleus in response to BL, and CRY2 function and degradation closely correlate with the formation of photobodies, which have been proposed as sites for CRY2 signal transduction and/or CRY2 degradation [35].

The CRYs are in an inactive monomeric state in the dark and potentially change to an active oligomeric state under BL [37]. The rate of CRY oligomerization reaction in light is much faster than that of the CRY monomerization in darkness. Photoactivation of CRYs begins with photoexcitation, which results in conformational changes and formation of the CRY homooligomers that interact with CRY-interacting proteins to alter gene expression and plant development [28]. During the process of photoactivation, either CRY1 or CRY2 undergoes blue-light-dependent phosphorylation to transduce the signal to the downstream component of the CRY signaling relay [35]. The phosphorylation of CRYs closely correlates with the intensity and duration of BL exposure [35]. This may partly explain why the effect of BL on plant flowering varies with the amount of light [8]. For example, BL can influence flowering only when delivered with sufficiently high intensities as night interruption (NI) lighting but is ineffective in regulating flowering at low intensities [19,20]. Additionally, photoactivation of CRYs can also be influenced by the presence of light wavelength(s) other than BL due to their interaction on the photoreceptors or cross-talk between photoreceptors [29,38]. This is supported by varying plant responses to blue LED in combination with other different LEDs [7,39,40,41,42].

Photoactivation of CRYs by BL can trigger a negative feedback reaction by increasing the BLUE-LIGHT INHIBITOR OF CRY (BIC) proteins to prevent excessive accumulation of active CRYs and maintain sustainable photosensitivity of the cell [28,43,44]. The BICs can inhibit blue-light-induced CRY1 activation, as well as BL-induced CRY2 degradation [28,44]. Also, the BICs appear to function only in BL, but not in other wavelengths, such as red light or FR light; however, the mRNA expressions of both *BIC1* and *BIC2* genes are induced by light in a wavelength-independent manner and are mediated by both CRYs and PHYs [43,45]. In the past, our lab has found that sole-source lighting with low- or modest-intensity blue LED light, which is associated with a low PPS value (low PHYB activity), resulted in low CRY1 activity but high CRY2 activity [38]. In this case, whether a balance of photoactivation and inactivation of CRYs is disrupted by the blue LED light through overexpression of the BIC due to co-action of CRYs and PHYs is unknown.

CRY2 is a major photoreceptor regulating BL-mediated photoperiodic flowering, but its effect on flowering varies with spectral quality (i.e., co-action of BL with other light wavelengths) and plant genotype. In *Arabidopsis*, CRY2 promotes flowering under long-day (LD) conditions [35]. However, in our recent study on *Arabidopsis* under constant sole-source lighting, the *cry2* mutant did not delay flowering under blue LED, but it did under blue + FR LED compared with wild plants [38]. Moreover, overexpression of *CRY2* did not affect flowering time under either blue or blue + FR LED. It appears that the role of CRY2 in BL control of flowering time is also affected by light spectral quality and, possibly, the expression of CRY2 can maintain a sufficiently activated level under blue + FR LED light [38]. In rice (*Oryza sativa* ), the knockdown of *CRY2*, but not of *CRY1*, delays flowering both in LD and short-day (SD) conditions [46]. However, a recent study indicates that overexpression of *CRY2* in rice with a photoperiod-insensitive genetic background does not affect the flowering time, although the *OsiCRY2 Arabidopsis* overexpressors exhibited early flowering [47], suggesting the effect of genetic background on the CRY2’s function. Furthermore, for tomatoes under LD conditions, *CRY2* overexpression delayed flowering [48].

CRY1 also plays a role in BL-mediated photoperiodic flowering, with the importance of the role varying with environmental conditions and plant genotype. In *Arabidopsis*, the *cry1* mutant exhibited a pronounced late-flowering phenotype at 16 °C, but not at 23 °C, suggesting that temperature influences the role of CRY1 in the photoperiodic regulation of flowering time [35,49]. It appears that the role of CRY1 in *Arabidopsis* flowering control is limited due to the evident effect only under specific environmental conditions [35]. In contrast to *Arabidopsis*, CRY1a rather than CRY2a in soybean (*Glycine max*) is a major regulator of photoperiodic flowering, and it exhibits circadian rhythmicity in varying photoperiods and strongly promotes floral initiation [46,50]. Also, sorghum (*Sorghum bicolor*) CRY1b can rescue the late-flowering phenotype of the *Arabidopsis cry1/cry2* double mutant, indicating a similar role to *Arabidopsis* CRY2 as a major regulator of photoperiodic flowering [46,51]. Additionally, in onions, overexpression of CRY1 leads to a significant acceleration of flowering time [36].

There are overlapping functions of CRY1 and CRY2 in regulating photoperiodic flowering. In tomatoes, both CRY2 and CRY1a likely function to repress flowering; the knockout of a single *CRY* gene, whether *CRY2* or *CRY1a*, does not affect flowering time, but the simultaneous knockout of both *CRY1a* and *CRY2* results in an earlier onset of flowering [46,52]. In *Arabidopsis*, the *cry1cry2* double mutant flowers later than either the *cry1* or *cry2* single mutants under LD or continuous BL conditions [53]. In our recent study on *Arabidopsis* under constant sole-source lighting with blue LED, neither the *cry1* nor the *cry2* mutant delayed flowering, but the *cry1cry2* double mutant inhibited flower initiation compared with wild plants [38]. The overlapping roles of CRY1 and CRY2 in regulating photoperiodic flowering are partly attributed to their shared downstream pathway components, such as COP1/SPA and PIF [54]. However, CIB is a specific downstream pathway component that accounts for CRY2′s roles in BL-mediated flowering, which is a key distinction between CRY1 and CRY2 [54]. Detailed information can be found in Section 4.1.

#### 2.1.2. Phytochromes (PHYs)

The PHYs are primarily photoreceptors of red and FR light, but they also perceive other light wavelengths, including BL, due to small absorption peaks [5,55]. Furthermore, PHYs are also able to sense changes in ambient temperature as well as day length and the difference between day and night [26,56,57].

In *Arabidopsis*, PHYs are encoded by five different genes (*PHYA* to *PHYE*) [22,57]. Most seed plants possess three PHYs: PHYA, PHYB, and PHYC [58]. PHYD and PHYE probably originated from duplications of PHYB in angiosperms and only in Brassicaceae, respectively [57]. However, some flowering plants lack PHYC (e.g., *Populus trichocarpa*) or PHYE (e.g., monocots) [58]. Several species, such as maize (*Zea mays*) and tomato, have two copies of PHYB, i.e., PHYB1 and PHYB2 [57,59]. Generally, in the majority of dicotyledonous plants, PHYs are encoded by five gene families (*PHYA* to *PHYE*), but, in monocotyledonous plants, PHYs are encoded by three genes, *PHYA*, *PHYB*, and *PHYC* [60]. PHYA is mainly activated by FR light, as well as BL [55], whereas PHYB-E are activated by red light and deactivated by foliar shade, as well as BL [22,61].

PHYs can be classified into different types or statuses according to certain features. Based on photolability, PHYs can be grouped into two types: type I photolabile forms and type II photostable forms [62]. PHYA is classified as a type I photolabile PHY and is unstable under light, but PHYB to PHYE are classified as type II PHYs and are relatively stable under prolonged light exposure [22,62]. Also, according to biological activity, PHYs exist in two forms: a biologically inactive red-light-absorbing form (Pr) and a biologically active FR-light-absorbing form (Pfr). The inactive Pr PHY is assembled in the cytosol, and, once converted to Pfr, PHYs move from the cytoplasm into the nucleus, where most signaling functions occur [22]. The inactive Pr state can partly photoconvert to the active Pfr state upon exposure to light (including BL), with the greatest conversion by red light, and the Pfr is partly inactivated under FR light or in the dark through a process of temperature-dependent thermal relaxation called thermal reversion [56,62].

Although BL can stimulate PHYs to some degree, it is less efficient than red light [63]. Plants grown under any light wavelengths have both Pfr and Pr, and the PHY activity can be indicated by the value of the phytochrome photostationary state [PPS; PPS = Pfr/(Pfr + Pr)] [64]. Active PHYB responses normally occur when PPS > 0.6, but active PHYA responses do so at much lower values, e.g., PPS < 0.2 [56,64]. The PPS value for plants growing under light can be calculated based on the light spectrum, and its absorption values can be calculated based on PHY [65]. Pure BL from narrow-band LED light normally has a low PPS value of around 0.5 depending on peak wavelength, but impure BL from traditional lighting sources, such as blue fluorescent light, has a high PPS value above 0.6 [66]. The blue LED in combination with a low level of red LED light increases the PPS value up to 0.7, but its combination with a low level of another light wavelength from UVA, UVB, green, or FR LED light still has a low PPS value below 0.6 [67,68].

The activation of PHYs induces conformational changes that trigger PHY-regulated responses [62,69]. In the activation process, the activity of PHYs greatly relies on their nuclear localization [22,62], and it is regulated by dephosphorylation and phosphorylation [70], as well as the formation of photobodies [22]. A photobody is an assembly of active PHY, Pfr, into large subnuclear compartments, which is a key step in PHY signaling [22]. Photobodies can act to enhance PHYB activity [22]. Red light increases Pfr stabilization and promotes photobody formation, while BL, as well as FR light, high temperature, and darkness, cause its disassembly [71]. This partly supports our lab’s reports that blue LED light causes low PHY activity [7,42].

A promotive role in flowering is played by PHYA. In *Arabidopsis*, the *phyA* mutant flowers later than wild type plants under LD and quasi-LD conditions, such as with night breaks or day extensions, and transgenic plants overexpressing *PHYA* flower earlier in both SD and quasi-LD conditions [72]. In *Arabidopsis*, PHYA, together with CRY1 and CRY2, also functions redundantly in mediating BL promotion of flowering. Monogenic *cry1*, *cry2*, or *phyA* mutants flowered at about the same time as the wild type, but a double mutant impaired in any two of the three photoreceptors or the *cry1cry2phyA* triple mutant flowered significantly later than the wild type when grown under continuous BL [73].

The PHYB generally inhibits floral initiation. *Arabidopsis phyB* mutant flowered earlier compared to the wild type in both LD and SD conditions, with a more pronounced effect under SD conditions, and similar flowering responses were observed in *phyB* mutations in pea (*Pisum sativum* L.) and sorghum [53,72]. This indicates that PHYB inhibits flowering in both LD and SD plants, with stronger inhibition in the photoperiod that typically suppresses flowering [72]. Despite its role as a floral inhibitor, PHYB’s function in floral initiation is likely more complex. For instance, transgenic *Arabidopsis* plants overexpressing *PHYB* also flowered earlier than the wild type, a phenomenon that is not easily explained [72].

The interaction between PHYA and PHYB demonstrates that PHYB inhibits flowering under both LD and SD conditions, a suppression necessary for the expression of PHYA’s promotive effect [74]. PhyA is exclusively responsible for promoting flowering through NI lighting, while PHYB plays a crucial role in detecting light quality during end-of-day light treatments, night breaks, and day extensions. Notably, PHYB functions differently and is subsequent to PHYA in controlling flower induction [74]. The subsequent role of the two photoreceptors was partly supported by a recent study on chrysanthemum, where plant flowering was not inhibited under prolonged photoperiod lighting with blue LEDs, despite increased expression of both PHYA and PHYB [16].

Besides PHYA and PHYB, other PHYs (PHYC/D/E) also play roles in plant flowering. PHYC plays distinct roles in the regulation of flowering time in different plant species [75]. In rice and *Arabidopsis*, PHYC appears to function as a flowering repressor under noninductive photoperiods [76,77]. However, in wheat (*Triticum aestivum*), PHYC promotes flowering under inductive photoperiods [75]. Like PHYB, both PHYD and PHYE inhibit photoperiodic flowering, but they play redundant roles with PHYB. The *phyD* or *phyE* mutant shows no changes in flowering time, unless in a *phyB* mutant background where flowering is earlier in double mutants [72]. PHYD, PHYE, and PHYB are also redundantly involved in mediating flowering as a shade avoidance response (SAR).

PHY seems to play a less important role in flowering’s response to BL compared to red light or white light, where an interaction between PHYs and CRYs is necessary. Our study on *Arabidopsis* indicated that a quintuple phytochrome mutant (*phyA phyB phyC phyD phyE*) did not change the flowering time under low-PPS BL (blue LED or 94% B + 6% R + FR LED), but it flowered earlier under high-PPS BL (94%B + 6% R LED) compared with wild type plants [78]. This suggests that PHY is not indispensable for BL-mediated flowering but contributes to the flowering response to BL under high-PPS background light conditions. However, under blue-containing light, such as white light, the interaction of PHYB and CRY2 is necessary to control fundamental processes (e.g., flowering time and circadian rhythm) in *Arabidopsis*, where the regulation of floral induction is mediated by the antagonistic actions of CRY2 and PHYB [53,63,79].

#### 2.1.3. ZEITLUPE (ZTL) Family Members

Another group of BL photoreceptors is ZTL family proteins [80,81]. ZTL family proteins are activated by illumination with BL; they undergo structural changes upon BL exposure that modulate protein activity [80,82,83]. ZTL family proteins transduce light signals primarily by altering the activity of the SCF E3 ligase complex [61,83]. ZTL family proteins did not show dark recovery as PHOT did in early studies [80], but later studies indicated that they did return to the dark state, despite slow adduct decay (occurring in the order of hours to days) [84,85].

The ZTL family comprises three proteins: ZTL protein, FLAVIN-BINDING, KELCHREPEAT, F-BOX (FKF1) protein, and LOV KELCH PROTEIN2 (LKP2) [81,82,83,86]. ZTL family proteins have an important role in the photoperiodic control of floral transition [81,82]. Despite the partly overlapping roles of these proteins, generally, ZTL exerts control over circadian rhythms, and FKF1 controls the transition to flowering, while LKP2 is required for both processes [83].

(1) ZTL

ZTL is a negative regulator of flowering, but it plays a predominant role in the control of the circadian clock, which can affect photoperiodic flowering [82]. ZTL itself is positioned in interconnected clocks in plants [61]. Also, ZTL plays some roles in the clock input of light by mediating the components of the circadian clock [87]. Furthermore, ZTL interacts with the clock component in a BL-enhanced manner, contributing to ZTL protein stabilization and accumulation by the end of the light period [82]. Additionally, ZTL photocycle kinetics are crucial in determining the circadian period length [82]. ZTL can sense BL fluence to mediate circadian timing [84]. ZTL mutants exhibit a fluence-dependent effect on the circadian period, resulting in a maximal circadian period of 36 h under low-light conditions (0.6–8 μmol m^−2^ s^−1^). Above 8 μmol m^−2^ s^−1^, the circadian period collapses to near wild type (28 h), indicating that ZTL circadian function may be limited to low-light conditions [84]. ZTL’s role in light intensity recognition suggests it is important in resetting the plant clock and contributes to the clock’s acceleration with increased light fluence [82]. ZTL also functions in temperature compensation of the circadian clock due to its involvement in warm-temperature responses [88]. Intriguingly, ZTL and CRYs play opposite roles in thermo-responses; the mechanism and potential physiological significance of this remain to be explored [88]. Although ZTL is a major protein within the group for circadian clock regulation, the ZTL homologs, LKP2 and FKF1, also contribute to controlling the pace and robustness of the circadian clock [89].

(2) FLAVIN-BINDING, KELCHREPEAT, F-BOX (FKF1)

FKF1 acts as a positive regulator of flowering in a light-dependent manner, particularly in the afternoon [61,87,89]. FKF1 plays a role in detecting LD signals and activating the photoperiodic flowering pathway [86]. BL seems to have a predominant role in FKF1’s function in flowering regulation [80]. Despite its transcripts oscillating with a circadian rhythm, FKF1 does not appear to have any intrinsic clock function but likely operates as part of the clock’s output [80]. Also, FKF1 is required for CRY-interacting factors, such as CIB3, to promote flowering in response to BL [90]. In *Arabidopsis*, FKF1 is activated by BL and promotes flowering under inductive LD conditions [91]. In rice, FKF1 not only has a similar role in photoperiod-mediated flowering relative to *Arabidopsis* but also promotes flowering independent of photoperiod [91].

(3) LOV KELCH PROTEIN2 (LKP2)

The LKP2 mediates both the circadian clock and flowering. Like ZTL, the LKP2 also plays a role in the regulation of the circadian clock by mediating the clock’s components [87]. The phenotypic parallelism observed in *ztl lkp2* double mutants compared to *ztl* mutants suggests that LKP2 functions in an intricate manner [87]. Also, LKP2, as well as ZTL, are involved in the suppression of flowering [92]. Although LKP2 does not function as a major regulator of the circadian clock and flowering time due to its low physiological expression, it binds the substrates of ZTL and FKF1 in vitro and partially contributes to the circadian clock and flowering [93]. Overexpression of LKP2 or ZTL, as well as deficiency of FKF1, result in late flowering in *Arabidopsis* under LD conditions [92].

#### 2.1.4. Other Photoreceptors

(1) Phototropins (PHOTs)

PHOTs act as BL photoreceptors, but they can also sense UVA light and temperature signals [81,83]. PHOTs are known for their central role in plant phototropism, but they can also indirectly contribute to plant flowering [94,95,96].

In the dark, PHOTs lack activity until the absorption of BL [83]. Multiple phosphorylation leads to dimerization and activation of PHOTs [83,94,97]. As a result, the activated PHOTs transmit this signal to downstream components within the signaling pathway [94]. However, in the dark, the photoreceptor reverts to the inactive state within several tens or hundreds of seconds [83].

Higher plants, including *Arabidopsis*, possess two isoforms of PHOTs: PHOT1 and PHOT2 [86,97]. BL triggers both PHOTs, but in different manners. It stimulates PHOT2 and downregulates PHOT1 [81]. For example, in *Arabidopsis* plants exposed to 3 h of lighting at 40 µmol m^−2^ s^−1^ after 16 h of dark adaption, both blue and red light downregulated PHOT1 transcript abundance but upregulated the PHOT2 level compared to the dark treatment [98]. Compared to red light, BL upregulated PHOT1, but it did not affect PHOT2 [98]. The PHOT1 is photostable, but PHOT2 is photolabile [83,97]. PHOT1 can be activated under much lower BL intensity than PHOT2 [80,97]. For example, under a BL of 10 µmol m^−2^ s ^−1^, over 85% of PHOT1 is activated, but only 40% of PHOT2 is activated [97]. Also, the dark reversion of PHOT2 is about 10 times faster than that of PHOT1 [80].

Limited information is available about the role of PHOT1 and PHOT2 in flowering mediated by BL. Our recent study on *phot* mutants in *Arabidopsis* has indicated that both PHOT1 and PHOT2 are partly involved in the promotion of plant flowering mediated by BL in association with low PHY activity [96]. However, it is still unclear whether the PHOTs directly or indirectly contribute to this process.

(2) Halotolerance protein (HAL3)

In rice, Sun et al. [99] found that OsHAL3, a highly conserved Flavin mononucleotide (FMN)-binding protein, serves as a new BL sensor. OsHAL3 is structurally inactivated by light, especially BL, through photo-oxidation and direct interaction with photons. Also, OsHAL3 has been identified as a positive regulator of flowering in rice; OsHAL3 overexpression lines exhibited an early flowering phenotype, whereas downregulation of OsHAL3 expression through RNA interference delayed flowering under an inductive photoperiod (i.e., SD conditions) [100]. OsHAL3 directly binds to the promoter of *Hd3a* and forms a complex with Hd1 to positively regulate flowering under SD conditions, especially at around 4 h before light onset, but the interaction is inhibited by blue or white light [100,101].

### 2.2. Photosynthetic Pigments

Despite not being protein-based receptors, photosynthetic pigments can indeed act as an additional group of receptors [25,102]. The photosynthetic pigments are essential for capturing and transferring light energy during photosynthesis [102]. Also, these molecules can respond to light signals and exert effects on plant physiology, including flowering [25]. The photosynthetic pigments mainly absorb photosynthetically active radiation (400–700 nm), although they can also absorb UVA with very low quantum efficiency due to the flavonoid shield [63]. Among PAR wavelengths, BL results in greater energy loss during the light reaction in photosynthesis than red or green light due to a higher frequency or energy [63]. Photosynthetic pigments may sense changes in BL quantity to affect photosynthesis and thus the availability of assimilates [21]. The photosynthetic pigments include the various chlorophylls and carotenoids [63].

#### 2.2.1. Chlorophylls (Chls)

The Chls are essential pigments required by all photosynthetic organisms to absorb light energy, and they play a key role in acclimation to environments with a varied light spectrum. Terrestrial plants only possess Chl a and Chl b. Chl a is the most abundant form of Chl in terrestrial plants, and Chl a has the highest absorption peak at 665 nm (red light) and is the main collector of light energy transferred to the photosynthetic reaction centers [63,103]. Chl b, with the highest absorption peak at 450 nm (BL), is only found in the antennae of the light-harvesting complexes [103]. Chl b has a critical role in harvesting light at lower light intensities as well as photoprotection [104]. A high Chl a/b ratio indicates greater acclimation to high light intensities and an enhancement in photosynthetic electron transport, whereas a low Chl a/b ratio indicates shade tolerance [103]. In many species, monochromatic BL causes a decrease in chlorophyll content and an increase in the Chl a/b ratio compared to those under multispectral light, despite a positive role of BL in combination with another wavelength (e.g., red light) [63].

There is no direct evidence of a relationship between chlorophyll accumulation and flowering time; however, studies on plants with altered chlorophyll metabolism showed that flowering time is changed compared to wild type plants [105]. In Chl b-less mutants, besides the imbalances in photosynthesis and growth, plants show a delay in the onset of flowering [104]. The complex pathways involved in regulating chlorophyll biosynthesis and breakdown may interact with the pathways regulating flowering [105]. However, as chlorophyll contents affect sugar biosynthesis [106], additional research will be required to disentangle the effects of chlorophyll and sugars in the regulation of the floral transition.

#### 2.2.2. Carotenoids

Carotenoids are principally thought to protect chlorophylls by quenching their triplet state via the transformation of excitation energy into heat [103]. Carotenoids absorb light strongly in the range of 480–580 nm, where chlorophylls have low absorption [25]. BL stimulates the accumulation of carotenoids in leaves when compared to red light, especially at higher irradiances (>200 μmol m^−2^ s^−1^), thus contributing to the leaf’s photoprotective capacity [63]. Carotenoids are less abundant than chlorophylls, with a carotenoid to chlorophyll ratio usually ranging from 0.1 to 0.5, depending on the species and environmental conditions [63,107].

Despite there being no direct relationship between carotenoid accumulation and plant flowering, Fibrillins (FIB/FBN/FIN), carotenoid-associated proteins, play a vital role in environmental-stress-induced flowering. It has been found that overexpression of pepper (*Capsicum annuum*) FIB in tobacco (*Nicotiana tabacum*) accelerated flowering when grown under high light [103].

### 2.3. Integration of Multiple Photoreceptors and Photosynthetic Pigments

There is a complex network of interactions between different photoreceptors, as well as between photoreceptors and photosynthetic pigments. For example, within different photoreceptors, CRY1 and PHYB appear to be the main photoreceptors involved in the regulation of *PHOT1* transcript accumulation, and the expression of *PHOT2* is dependent on both CRYs and PHYA [98]. The interactions between photosynthetic pigments and photoreceptors are implied by significant overlapping of their absorption spectra [94]. Also, the interactions can be attributed to the fact that photosynthesis mediated by photosynthetic pigments requires coordinated regulation of the nuclear and plastidic genomes [81]. On one hand, a variety of intermediates from chloroplastic metabolic pathways, such as carbohydrates and reactive oxygen species, have been identified as signals emitted by the chloroplast to deliver information to the nucleus to affect photoreceptor action [108]. On the other hand, the expression of numerous nuclear genes involved in plastid transcription and photosynthesis is controlled by CRYs in response to BL [24].

The final expressions of different photoreceptors and the accumulation of photosynthetic pigments under BL are the results of their interactions. For *Arabidopsis* grown under sole-source lighting at a PPFD of 120 µmol m^−2^ s^−1^ and a photoperiod of 16 h, plants had earlier flowering under blue LED than white LED and white fluorescent light [109]. In this case, blue LED significantly reduced *PHYA*, *PHYD*, and *CRY1* mRNA levels and slightly decreased *PHYB*, *PHYE*, and *PHOT1* mRNA levels but did not change the mRNA levels of *PHYC*, *CRY2*, and *PHOT2* compared with the white fluorescent light. Blue LED did not affect mRNA levels of *PHYD*, *CRY2*, and *PHOT2* but reduced those of other photoreceptors compared with white LED. The reduction of photoreceptor gene expression manifests the reduced sensitivity of light-sensing molecules and their regulatory properties toward BL [110]. Also, plants under blue LED and white LED had similar content of the main photosynthetic pigments per dry weight unit, but they had lower content than those under white fluorescent light [109].

Despite interaction between multi-photoreceptors, it is possible that the contribution of different photoreceptors to BL-mediated flowering varies with plant species. For three SD plants, chrysanthemum, perilla (*Perilla frutescens*), and kalanchoe (*Kalanchoe blossfeldiana*), NI lighting with BL did not inhibit flowering in chrysanthemum, but it did in perilla, and BL had a secondary effect on the control of flowering of kalanchoe while red light played the main role [8,111,112]. Possibly, two light-regulating systems through PHY and CRY function at the same time in perilla, but only one system (PHY) functions in chrysanthemum, and a greater role was played by PHY than CRY in kalanchoe [112]. In *Phalaenopsis* orchids (*Phalaenopsis* spp.), whether spiking is improved by daytime supplemental lighting depends on the relative amount of active PHY [113].

Additionally, the contribution of different photoreceptors to BL-mediated flowering varies with other light wavelength(s) co-acted with BL. This is supported by the impact of shifts in the spectral quality of light on gene expression in chrysanthemums by NI lighting with blue and other LEDs [114]. The NI lighting sequentially with FR and blue LED promoted flowering associated with higher expression of flowering inducer genes *PHYA* and *CRY1*. However, NI lighting sequentially with blue and red light failed to induce flowering due to higher expression of *PHYB* and *ANTI-FLORIGENIC FT/TFL1 FAMILY PROTEIN (AFT)*, despite high expression of *CRY1* and *FLOWERING LOCUS T-LIKE (FTL)*. It appears that the co-action of BL with different light wavelengths can change the balance of both photoreceptors and floral integrator proteins, where the FTL and AFT positively and negatively regulate flowering, respectively.

## 3. Floral Integrator Proteins

**Figure 2:**
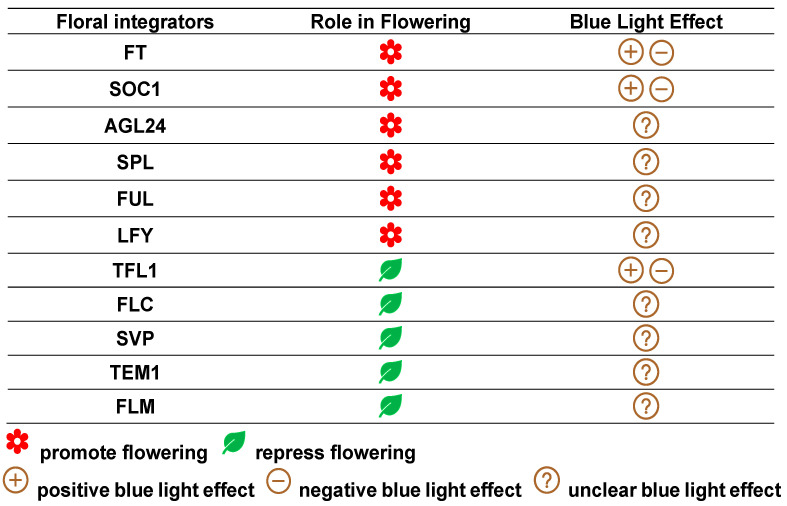
An illustration of the characterization of floral integrator proteins in terms of their role in flowering and their response to blue light. FT = FLOWERING LOCUS T; SOC1 = SUPPRESSOR OF OVEREXPRESSION OF CONSTANS 1; AGL24 = AGAMOUS-LIKE 24; SPL = SQUAMOSA PROMOTER BINDING-LIKE; FUL = FRUITFULL; LFY = LEAFY; TFL1 = TERMINAL FLOWER 1; FLC = FLOWERING LOCUS C; SVP = SHORT VEGETATIVE PHASE; TEM1 = TEM-PRANILLO1; and FLM = FLOWERING LOCUS M. If both positive and negative effects of blue light are shown together, this indicates the blue light effect may vary with plant genotype and the application of blue light.

Floral integrators are specifically a group of genes that act as “decision-makers” for flowering because they function as “hubs” in the flowering network by integrating environmental and intrinsic signals that are sensed by different upstream pathways [115]. These floral integrators can be roughly grouped into two types in terms of their function in floral induction: positive floral integrators (the proteins they encode act as floral activators) and negative floral integrators (the proteins they encode act as floral inhibitors) (Figure 2).

### 3.1. Floral Activators

The major floral activators in *Arabidopsis* include FLOWERING LOCUS T (FT), SUPPRESSOR OF OVEREXPRESSION OF CONSTANS 1 (SOC1)/AGAMOUS-LIKE 20 (AGL20), AGAMOUS-LIKE 24 (AGL24), SQUAMOSA PROMOTER BINDING-LIKE (SPL), FRUITFULL (FUL)/AGL8, LEAFY (LFY), and other relevant regulators [115,116]. FT exerts a positive influence on SOC1, with this FT-to-SOC1 module occupying a central role in plant flowering, exhibiting evolutionary conservation across diverse plant species [117]. In *Arabidopsis*, FT emerges as a direct downstream target of both CONSTANS (CO) within the photoperiod pathway and FLOWERING LOCUS C (FLC) in the vernalization/autonomous pathway [115]. SOC1, on the other hand, is under the direct sway of SPL in the aging pathway, alongside FLC and DELLA proteins within the gibberellin (GA) pathway [117]. Therefore, FT and SOC1 are the two key components among the major floral activators.

#### 3.1.1. FLOWERING LOCUS T (FT)

FT is the earliest identified florigen, a floral activator, in *Arabidopsis* [116]. Florigen is ubiquitous in flowering plants, but the number and name of FT homologs vary with plant species [118], and all *FT* homologous genes within each species do not play an equally important role in flowering regulation [119]. For example, specific major FT homologs regulating flowering in other crops include HEADING DATE 3a (Hd3a) and RICE FLOWERING LOCUS T 1 (RFT1) in rice, ZEA MAYS CENTRORADIALIS 8 (ZCN8) in maize, GLYMA.16G150700 (GmFT2a) and GLYMA.16G044100 (GmFT5a) in soybean, SINGLE-FLOWER TRUSS (SFT) in tomato, and FLOWERING LOCUS T-LIKE 3 (FTL3) in chrysanthemum [118,120,121]. In rice, Hd3a and RFT1 are essential for promoting rice flowering under SD conditions, while RFT1 functions as a floral activator under LD conditions [119].

In *Arabidopsis*, FT is produced in the vascular leaf bundle and transferred via the phloem to the shoot apical meristem (SAM) to induce the floral transition [118]. In the SAM cells, FT binds to a 14-3-3 protein in the cytoplasm, enters the nucleus, and interacts with FLOWERING LOCUS D (FD), creating a florigen activation complex (FAC) [118,122,123]. The FAC then induces the expression of floral meristem identity genes, such as *APETALA 1 (AP1)*, *SOC1*, and *FUL*, leading to floral induction [26]. In rice, similarly to *Arabidopsis* FT, both Hd3a and RFT1 are expressed in the vascular tissue of leaves and move to the SAM, where they enhance the expression of floral meristem identity genes, such as *OsMADS14* and *OsMADS15*, and trigger flowering [119,124,125].

BL’s effect on *FT* gene expression varies with plant species, lighting source, lighting duration, and time of the day, which is associated with varying flowering responses to BL. In *Arabidopsis* under BL, CRY2 binds with CIB proteins, which directly bind to the promoter of *FT* and promote its transcription [54,126]. In day-neutral-type strawberries (*Fragaria × ananassa*), FvFT1 was induced by blue LED light and exclusively expressed in the veins of older leaves [127], which may partly explain the promotional effects of blue LED light on the number of flower clusters and final strawberry yield [128]. In sweet pepper (*Capsicum annuum*), sole-source daytime lighting with blue LED light induced the expression of CaFT1 and CaFT2 and caused earlier flowering compared with white light [129]. In rice, NI lighting with impure BL suppressed Hd3a expression through activating PHYB and delayed flowering [130]; however, blue LED light in the morning induced Hd3a expression to promote flowering by activating Ehd1 expression [131]. In petunia, PehFT expression was higher on the first day but lower on the seventh day under blue vs. red LED light, suggesting that it may not be the major contributor to BL-promoted flowering in this species [132].

#### 3.1.2. SUPPRESSOR OF OVEREXPRESSION OF CONSTANS 1 (SOC1)

SOC1 plays regulatory roles in integrating multiple flowering signals for floral transition and reproductive development in *Arabidopsis* [133]. SOC1 interacts with AGL24 to promote the differentiation of primordia into floral meristems via upregulating LFY [134]. SOC1 expression is directly regulated by FT positively and by FLC negatively [115,134]. The role of SOC1 in floral induction has also been found in other species, such as cotton (*Gossypium hirsutum*) [133]. Also, the *Brassica rapa* genome possesses three *SOC1* genes, and *BrSOC1-1* and *BrSOC1-2* function redundantly in flowering time regulation [115].

The BL effects on SOC1 and other floral integrator proteins vary with plant species, cultivars, and reference light. Prolonged photoperiod lighting with BL promoted flowering through the induction of FT and SOC1 expression in *Arabidopsis* but not in another LD plant, *Gypsophila paniculata*, where both GpFTs and GpSOC1 expressions were low with BL induction [14]. In *Gypsophila paniculata*, two cultivars showed different expressions of GpFT and GpSOC1, which was associated with their contrasting flowering responses to blue light [11]. In petunia, *FLORAL BINDING PROTEIN 28 (FBP28)*, a SOC1-like gene, not only had a role in transmitting the BL signal from the FT protein to induce floral bud formation but also increased expression under blue LED light compared with red LED light [13]. In LD plant lisianthus (*Eustoma grandiflorum*) under SD conditions, overnight lighting with low-intensity blue LEDs promoted flowering compared to no lighting, and this is associated with increased expression of *EgFTL* and *EgSOC1L* [135]. However, later and lower expression of these genes was induced by blue LED compared to FR LED, suggesting weaker promotion of flowering by BL than FR light in lisianthus.

#### 3.1.3. Other Floral Activators

Despite the existence of some other floral activators, limited information is available about their response to BL. In *Arabidopsis*, the expression of an *SPL* gene, *AtSPL8*, is positively regulated by CRYs [136]. However, whether blue LED lighting affects its expression associated with the flowering response is unknown.

### 3.2. Floral Inhibitors

The major floral inhibitors in *Arabidopsis* include TERMINAL FLOWER 1 (TFL1), FLOWERING LOCUS C (FLC), SHORT VEGETATIVE PHASE (SVP), TEMPRANILLO1 (TEM1), and FLOWERING LOCUS M (FLM) [120,137,138,139]. These negative regulators act antagonistically to positive regulators by controlling parallel signaling pathways [26]. For example, FLC acts with its partner SVP to repress transcription of the flowering-promoting genes *FT*, *FD*, and *SOC1* [115]. TEM1 acts within the leaf to repress FT, while FLC works both within the leaf and in the SAM, acting on different targets [26]. SVP delays the floral transition by regulating *SOC1*, *FT*, and *FLC* genes [139]. FLM interacts with SVP and forms a complex to prevent flowering [120]. Among these floral inhibitors, information about their involvement in BL-mediated flowering response is available for only TFL1 so far.

TFL1 is a closely related FT homolog, and it has been identified as an anti-florigen, a floral inhibitor, in *Arabidopsis* [118]. From other crops, the identified anti-florigens include ANTI-FLORIGENIC FT/TFL1 FAMILY PROTEIN (AFT) in chrysanthemum, RICE CENTRORADIALIS (RCN) in rice, and SELF PRUNING (SP) in tomatoes [26,118,120,121,140].

The BL effect on TFL1 may vary with plant species, even from the same photoperiod response group. In SD plant chrysanthemum, AFT (CsAFT) is induced by the coincidence of PHY signals (i.e., activated PHYB) with the photosensitive phase set by the dusk signal under non-flowering-inductive conditions (e.g., NI lighting with red light and LD conditions) [141]. Plant flowering occurred only when night length exceeded the photosensitive phase for CsAFT induction. This may partly explain why prolonged photoperiod lighting with blue LEDs did not prevent flowering in this species [15,16,17,18], as PHYB cannot be activated by blue LEDs to induce AFT. Differing from chrysanthemum, in SD-type strawberry, end-of-day (EOD) lighting with BL or FR light could induce greater expression of FvTFL1, the repressor of floral induction [142].

### 3.3. Co-Action of Floral Integrator Proteins

In *Arabidopsis*, FT and TFL1 function oppositely and play antagonistic roles in the determination of flowering time [120,143]. TFL1 forms a complex with FD and acts to repress flowering by modulating FD-dependent transcriptional regulation [120,143]. In rice, RCN (a TFL1 homolog) inhibits flowering by competing with Hd3a (a FT homolog) for 14-3-3 binding to form a florigen repression complex (FRC), and the balance between FRC and the florigen activation complex (FAC) regulates the development of the SAM [118,144]. Similarly, other FT/TFL1-like proteins, such as SFT/SP in tomatoes and FTL3/AFT in chrysanthemum, act as activators/repressors of flowering and determine the timing of flowering in coordination [120,121,145].

BL can affect the balance of floral integrator proteins as well as other flowering regulators. In strawberries, associated with promoted flowering by blue LED light in LD conditions, the expression of an LD-specific floral activator FaFT1 was stimulated, while that of a flowering suppressor FaTFL1 was inhibited, resetting the balance of expression between these two opposite flowering regulators [146]. In kalanchoe, supplementing white LEDs with low-intensity blue LEDs at the end of the day increased flower bud formation under both SD and LD conditions [147]. This was associated with higher expression of the flowering promoter genes (*KfPHYA*, *KfCRY1*, *KfFT*, and *KfFPF-1*) and lower expression of the flowering suppressor gene (*KfPHYB*).

The BL application method, photoperiod, and leaf age can affect flowering by modulating both positive and negative floral integrator proteins (as well as other flowering regulators) in chrysanthemums. For example, when two light wavelengths were sequentially applied, the NI lighting treatments ended with BL (NI-RB and NI-WB) induced flowering, but those that ended with red or white light (NI-BR and NI-BW) did not [114]. For the latter treatments, despite high levels of *CRY1* and *FTL* gene expression, there was also greater expression of flowering inhibitor genes, like *PHYB* and *AFT*, suggesting that the balance of overall expression patterns of the above genes affects the flowering response [110]. Also, chrysanthemum flowering was promoted by exposure to low-intensity blue LEDs as either 4 h EOD SL or NI under SD conditions but only as 4 h EOD SL under LD conditions [148]. The flowering-promotion treatments were associated with greater gene expression of the florigen gene *CmFTL3*, floral meristem identity genes *APETALA1 (CDM111)*, *FRUITFULL (CmAFL1)*, and *LEAFY (CmFL)*, and two photoreceptor genes, *CmPHYA* and *CmCRY1*, but lower expression of anti-florigenic gene *CmAFT* and the photoreceptor gene *CmPHYB*. Additionally, the youngest leaf showed greater sensitivity to BL, influencing gene expression more effectively than other parts [148].

## 4. Signal Transduction Pathways

Light, as one of the environmental cues, regulates flowering through quality, quantity, and duration. From light receptors to floral integrator proteins, there are at least three signal transduction pathways for flowering mediated by light (including BL): the photoperiod pathway, the light quality pathway (or shade pathway), and the light quantity pathway (or photosynthesis pathway) [21]. For some species, the BL seems to promote flowering as a strong stimulus or signal over the critical day length [149], which implies that BL can affect plant flowering initiation in more than the photoperiod pathway, at least for these species. Intensive molecular genetic and genomic studies in *Arabidopsis* have provided significant insights to distinguish the three pathways for flowering regulation, despite many cross-talks between these pathways, as well [21].

### 4.1. Photoperiod Pathway

**Figure 3:**
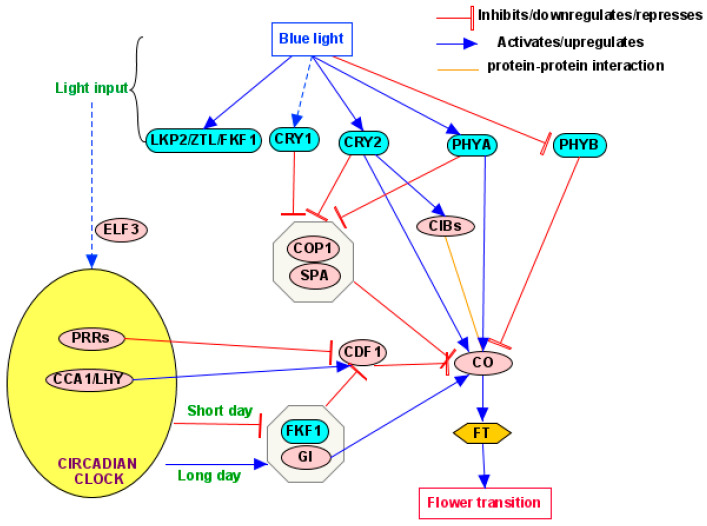
A simplified diagram of the photoperiod pathway in blue-light-mediated floral transition in *Arabidopsis thaliana*. FT = FLOWERING LOCUS T, a florigen; CO = CONSTANS, a central transcription factor in photoperiod pathway. The involved main blue light photoreceptors include CRY1/2 = cryptochrome 1/2; PHYA/B = phytochrome A/B; ZTL = ZEITLUPE, FKF1 = FLAVIN-BINDING, KELCHREPEAT, F-BOX1; and LKP2 = LOV KELCH PROTEIN2. The involved key circadian clock components include GI = GIGANTEA, an important clock output member; ELF3 = EARLY FLOWERING 3, an important clock input member; PRRs = PSUEDO RESPONSE REGULATORs, core clock components; and CCA1/LHY = CIRCADIAN CLOCK ASSOCIATED 1/LATE ELONGATED HYPOCOTYL, two core clock components. Other involved key transcription factors include CDF1 = CYCLING DOF FACTOR1; CIBs = CRYPTOCHROME-INTERACTING basic helix–loop–helixes; and COP1/SPA = CONSTITUTIVE PHOTOMORPHOGENIC1/SUPPRESSOR OF PHYA-105. Each gray hexagon with two transcription factors inside indicates a protein complex. Under long day conditions, GI and FKF1 form a complex, leading to the degradation of CDF and thus an increase in CO transcript abundance; however, under short day conditions, the expression of GI precedes that of FKF1, which disrupts the formation of the GI–FKF1 complex and thus reduces the abundance of CO [151]. The dashed lines indicate that the action can be affected by many factors or the involved detailed action mechanism is still unclear. To be clear, only the key pathway components are presented in the diagram, and information about other pathway components can be found in the manuscript’s text. Also, the regulations at the transcriptional and translational levels are not distinguished in the figure for simplification, and the relevant information can be found in the text.

The photoperiod pathway controls flowering in response to day length and especially night length [5]. In this pathway, photoreceptors entrain and interact with the circadian clock to activate clock-associated genes at different times of the day, enabling plants to measure photoperiod length [21,137,150]. In turn, the circadian clock regulates the rhythmic expression of downstream flowering genes, such as *GIGANTEA (GI)*, *CO*, and *FT* [5,21,137] (Figure 3).

In the photoperiodic flowering pathway, both PHYs and CRYs act as main photoreceptors, with ZTL and FKF1 acting as accessory photoreceptors contributing to clock entrainment and signal integration [21,150]. Specifically, in the photoperiodic pathway of BL-mediated flowering, CRY1 and CRY2 are the primary photoreceptors, supported by FKF1 and ZTL [5]. PHYA and PHYB also participate in BL signaling and are known to directly and physically interact with CRYs [95]. In *Arabidopsis*, photoperiodic flowering is primarily regulated by the expression of FT, which acts as a florigen [21]. The detailed pathway from photoreceptors to FT may vary with plant species. Taking the PHYs’ pathway as an example, the regulation of photoperiodic flowering in soybeans acts through PHYA-LUXE1-FT and is different from the PHYB-CO-FT flowering pathway in many nonlegume plants [150,152]. Also, normally, there is more than one pathway from a certain photoreceptor to FT. In *Arabidopsis*, CRY-mediated photoperiodic flowering follows at least three main pathways [35]. (1) Entrainment of the circadian clock by CRY1 and CRY2 and modulation of CO expression [153]. (2) CO protein stabilization by CRY1 and CRY2 (while CRY2 plays a primary role) under LD conditions [35]. (3) Activation of CIB transcription factors by CRY2 to promote FT expression [126,154,155]. Among the numerous components in the photoperiodic flowering pathway, CO is a central regulator of FT expression in *Arabidopsis* [156]. Normally, CO has a positive role in flowering induction in LD; CO binds directly to the *FT* promoter, upregulating FT expression [156,157]. In *Arabidopsis*, CO is regulated by the integrated signals from the circadian clock, day length, and light quality [115,157]. Due to the mediation of the circadian clock, the CO transcription level varies daily, with a minimum in the morning and a maximum in the evening under LD conditions [115]. Meanwhile, CO protein is stabilized in LD conditions but degraded in SD conditions [156]. Under LD conditions, CO protein accumulation coincides with increased CO transcripts in the afternoon to promote FT activation and floral transition [115]. Additionally, CO protein accumulation is regulated by light quality through photoreceptors [115]. In the dark, CO is degraded due to the COP1/SPA1 complex [26]. In the morning to noon, CO is degraded by CYCLING DOF FACTORS (CDFs) in a PHYB-dependent manner due to the greater red light percentage, while in the afternoon to evening, CO is gradually stabilized by the photoreceptors PHYA, FKF1, and CRY2 due to the gradually increased percentage of blue and FR light [26,60,119,158].

It is well-established that CDFs are key factors in the photoperiod pathway in *Arabidopsis* by controlling expression patterns of key flowering regulators, such as CO and FT [159]. In *Arabidopsis*, the CDFs can repress CO and FT expression redundantly and delay the flowering time [160]. In particular, CDF1 negatively regulates CO in the morning by interacting with the TOPLESS (TPL) co-repressor [137,161]. CDF protein stability is reduced in LD afternoon due to the degradation by a GI–FKF1 protein complex [159]. Furthermore, the gene expression level of *CDF1* is regulated sequentially from morning to evening by the circadian clock’s core components [160].

The GI serves as a key mediator between the circadian clock and the master flowering regulators (CO and FT) in the photoperiod pathway. GI is a gating factor in the output pathway of the circadian clock [162]. Also, GI promotes flowering by increasing the expression of CO and thus FT [163]. Under LD conditions, GI and FKF1 form a complex, leading to the degradation of CDF and thus the increase in CO transcript abundance; however, under SD conditions, the expression of GI precedes that of FKF1, which disrupts the formation of the GI–FKF1 complex and thus reduces the abundance of CO [151]. In addition to the CO-dependent regulation of FT, GI can independently regulate FT [164,165].

In addition to GI, the core circadian clock components also mediate daily variations of CO levels. Upon sensing the light of dawn, the core clock components CIRCADIAN CLOCK ASSOCIATED 1 (CCA1) and LATE ELONGATED HYPOCOTYL (LHY) activate the CDF to repress CO transcription [115]. During the afternoon and evening, CDF expression is repressed by the clock component PSUEDO RESPONSE REGULATORs (PRRs) [115]. Also, the PRR proteins directly stabilize CO proteins; PRR9 contributes to CO accumulation in the morning, whereas PRR5, PRR7, and TOC1 (PPR1) function in the evening [156,166]. Additionally, PRRs and FKF1 can form a protein complex and repress the transcription of CDFs to stabilize CO proteins [156,167].

As a key component of the input pathway of the circadian clock, EARLY FLOWERING 3 (ELF3) shows sensitivity to photoperiod changes and is regulated by the circadian clock while mediating flowering by affecting GI and CO levels [168,169,170]. Increasing day length increases the nuclear accumulation of ELF3, but increased darkness causes the degradation of ELF3 to an undetectable level [170]. In *Arabidopsis*, the *ELF3* genes are highly expressed during the evening time and downregulated in the early morning [168]. ELF3 promotes the degradation of GI through COP1, thus repressing flowering [171]. ELF3 and CO interact by preventing CO from building up in the late afternoon and early evening and causing late flowering in plants [168]. So, the opposing expression and regulation of ELF3 and CO work together to create a finely tuned regulatory loop, allowing for optimal flowering time [168].

Several signaling hub components convey BL signals from photoreactors to downstream components. CONSTITUTIVE PHOTOMORPHOGENIC1/SUPPRESSOR OF PHYA-105 (COP1/SPA) acts as a key repressor of light signaling downstream of photoreceptors [95]. COP1/SPA is active primarily in darkness [172], but, upon BL perception, photoreceptors (such as CRYs) directly interact with the COP1/SPA complex to suppress its activity [95]. A pivotal function of COP1/SPA in the regulation of flowering time by photoperiod involves the transcription factor CO [172]. In darkness, CO is degraded via COP1/SPA, which causes a delay in *Arabidopsis* flowering under non-inductive SD conditions and therefore allows for the adjustment of flowering time to seasons [172]. COP1 also suppresses flowering by promoting the degradation of GI, a circadian-clock-associated protein, in an ELF3-dependent manner, and it functions as an integrator of photoperiod and circadian signals [153]. In addition to COP1, the other two transcription factors, PIFs and HY5, can also convey BL signaling perceived by photoreceptors to regulate the circadian clock or directly to mediate floral integrator genes to affect flowering. Detailed information can be found in Section 4.2 and Section 5.3.

Despite the above general pathway, the specific involvement of the pathway components may be different depending on the methods of applying BL to plants. For example, at the end of the day, in response to BL, CRY2 interacts with SPA and suppresses COP1 activity, which decreases CO degradation and promotes FT expression [5]. When sensing BL under LD conditions in *Arabidopsis*, FKF1 interacts with GI, and the resulting FKF1–GI complex mediates BL-dependent degradation of CDF proteins that repress the transcription of *CO* and *FT* genes [5,173]. FKF1 also interacts physically with CO in a BL-dependent manner, and the FKF1-CO complex increases CO stability in LD conditions [174]. When BL is applied at the beginning of the day, PHYA may play a role in the plant’s responses. PHYA has been identified as a sensitive detector of both dawn and short photoperiods [175]. PHYA protein accumulates during the night, especially in short photoperiods; at dawn, PHYA activation by light results in a burst of gene expression, with consequences for physiological processes [175].

### 4.2. Light Quality/Shade Pathway

Flowering is accelerated, besides increased elongation growth, for plants growing under shade conditions, which is normally indicated by altered light quality, such as decreased R/FR ratios and lower BL levels [21,176]. This light-quality-regulated flowering response is actually a shade-avoidance response (SAR), which has a pathway independent of photoperiodism [21]. In this case, the light quality pathway for flowering regulation can also be called the shade pathway.

For the shade pathway, CRY1 seems to be the most important photoreceptor involved in BL-mediated flowering, despite unignored contributions from PHYs (especially PHYB). PHYB is a major photoreceptor in the SARs induced by decreased R/FR ratios, but there is redundancy with PHYD and PHYE [21,177]. A characteristic of this pathway is that the early flowering phenotype in the absence of PHYB is temperature sensitive, and the sensitivity lies in a flowering-specific branch of the PHYB signaling pathway rather than other aspects of SARs [21]. In addition, PHYA is a positive regulator of shade-regulated flowering time [177], although PHYA can also inhibit SAS in deep shade conditions, where FR light is much enriched and dominant [178]. CRY1 is the primary photoreceptor controlling the low-BL-induced SAR in adult plants [35]. There is a synergistic interaction between PHY and CRY signals, and the presence of both of these signals has more severe effects than either of these alone [179]. Due to a low PPS value, continuous sole-source lighting with blue LED at low to modest intensity can induce low activity of both PHYB and CRY1 [38,78]. This might accelerate flowering as one of the SARs, regardless of the photoperiodism type of the plant species [7].

**Figure 4:**
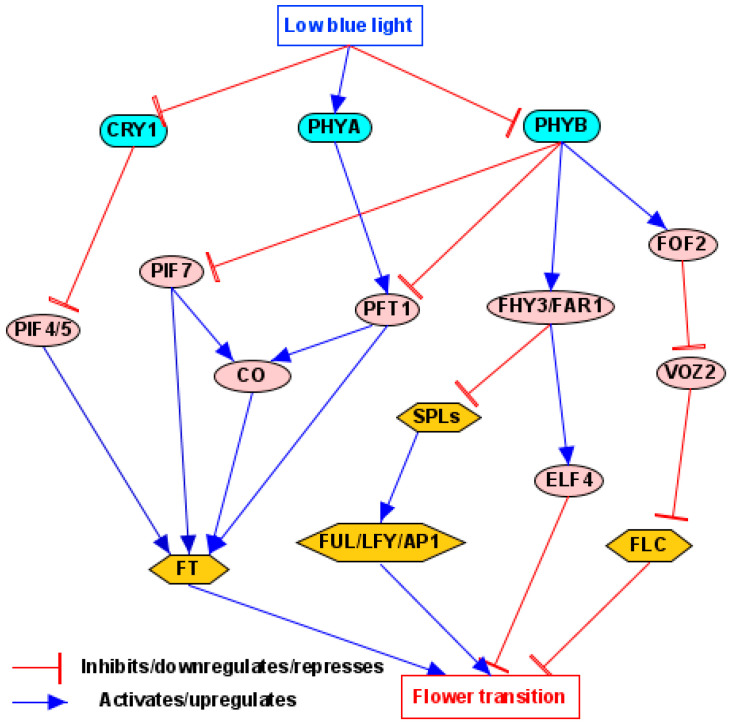
A simplified diagram of the light quality pathway (or shade pathway) in blue-light-mediated floral transition in *Arabidopsis thaliana*. The involved floral integrator proteins include FT = FLOWERING LOCUS T, a florigen; SPL = SQUAMOSA PROMOTER BINDING-LIKE; FUL/LFY/AP1 = FRUITFULL/LEAFY/APETALA1; and FLC = FLOWERING LOCUS C. The involved main blue light photoreceptors include CRY1 = cryptochrome 1; and PHY A/B = phytochrome A/B. The involved key transcription factors include PIF4/5/7 = PHYTOCHROME INTERACTING FACTOR4/5/7, an important group in the shade pathway; CO = CONSTANS, a central component in the photoperiod pathway; ELF4 = EARLY FLOWERING 4, an important clock component; PFT1 = PHYTOCHROME AND FLOWERING TIME 1; FHY3/FAR1 = HYPOCOTYL3/FAR-RED IMPAIRED RESPONSE1; FOF2 = F-box of Flowering 2; and VOZ2 = VASCULAR PLANT ONE-ZINC FINGER 2. To be clear, only the key pathway components are presented in the diagram, and information about other pathway components can be found in the manuscript’s text. Also, the regulations at the transcriptional and translational levels are not distinguished in the figure for simplification, and the relevant information can be found in the text.

Like the photoperiod pathway, shade-pathway-regulated flowering in *Arabidopsis* also mainly acts through the regulation of the florigen, FT [21,132] (Figure 4). This was confirmed by studying the effect of shade on flowering acceleration in 1001 *Arabidopsis* accessions, revealing that natural variation within the FT locus is the primary cause of shade-induced flowering [26,180]. The photoreceptors act through FT, implying that the shade pathway is leaf-based, which is supported by the fact that FT expression was suppressed by PHYB expressed in mesophyll cells [181].

PIFs are very important components in the shade pathway, and, for flowering regulated by shade, the most common pathway is PHY/CRY-PIFs-FT. Shade causes the inactivation of PHYB and/or CRY1, leading to the accumulation of PIFs, and an increase in PIF abundance leads to the induction of FT and its close homolog TWIN SYSTER OF FT (TSF) [26,182]. Among the PIF members, PIF4, PIF5, and PIF7 play a predominant role in shade-induced flowering by directly upregulating FT/TSF while downregulating Pri-miR156E/F [182]. It is worthwhile to note that different PIFs are involved in the CRY- and PHY-mediated SAR [35], as PIF4/5 predominantly regulates the CRY-mediated SAR but PIF7 exerts the predominant effect on the PHY-mediated SAR [35,176]. There is an antagonistic relationship between the photoreceptors and PIFs. Taking the relationship between CRY1 and PIF4/5 as an example, when BL is abundant, CRY1 suppresses the action of PIF4/5 by directly binding to them; however, in low BL conditions under shade, this repression is lifted, and the PIFs are able to promote SARs [179].

In addition to PIFs, the CO is also partly involved in the flowering regulated by shade in the PHY-FT pathway. Shade-triggered flowering involves the promoted expression and protein stability of CO, which acts to induce the expression of FT and TSF [182]. CO together with PIF7 plays an additive role in the promotion of flowering under shade conditions; however, LONG HYPOCOTYL IN FAR-RED1 (HFR1), a negative regulator of SAR response, is involved in the interaction with and inactivation of CO and thus the suppression of flowering, balancing the action of PIF7 [156,177,183]. Additionally, PHYA likely affects flowering time by regulating the protein stability of CO, showing a function independent of PIF7 [177]. It has also been found that CO contributes to shade-induced FT activation synergistically with the ATP-dependent chromatin remodeling factor, PICKLE [26,184].

PHYTOCHROME AND FLOWERING TIME 1 (PFT1) is also involved in flowering regulation by shade in the PHYB-FT pathway [21]. PFT1 is essential for PHYB regulation of flowering time [185] and primarily regulates flowering downstream of PHYB in a photoperiod-independent pathway [158]. PFT1 is equally able to promote flowering by modulating both CO-dependent (PHYB-PFT1-CO-FT) and CO-independent (PHYB-PFT1-FT) pathways, supported by the role of PFT1 as an activator of CO transcription and also FT transcription in a CO-independent manner [186]. Although PFT1 also regulates floral transition downstream of PHYA [55], this may be an indirect effect of changes in the PHYB pathway due to interaction between PHYA and PHYB [185].

Besides the PHYB-FT pathway, another pathway, PHYB-SPLs-FUL/LFY/AP1, is also involved in flowering regulated by shade. For this pathway, FAR-RED ELONGATED HYPOCOTYL3 (FHY3) and FAR-RED IMPAIRED RESPONSE1 (FAR1) act downstream of PHYB/D/E and are positively regulated by these photoreceptors [182,187]. Meanwhile, FHY3 and FAR1 interact with SPL3/4/5, flowering-promoting TFs, and inhibit their binding to key flowering regulatory genes, such as *FUL*, *LFY*, *AP1*, and miR172C, thereby delaying flowering [182]. Shade decreases FHY3 and FAR1 levels and prevents the above process, leading to early flowering [188]. Additionally, FHY3 and FAR1 delay flowering time under both LD and SD conditions by transcriptionally upregulating Early Flowering4 (ELF4) [189], and they were shown to interact with PIF3/5 and act as negative regulators of SAS [182].

Recently, the PHYB-FLC pathway has also been identified to regulate flowering by shade. In this pathway, F-box of Flowering 2 (FOF2) functions downstream of PHYB to promote FLC expression and inhibit flowering under simulated shade conditions, which are partially dependent on VASCULAR PLANT ONE-ZINC FINGER 2 (VOZ2) proteins [190]. Under the mimic shade, PHYB mediates the stabilization of FOF2 and leads to the degradation of VOZ2. This suggests a novel mechanism whereby plants fine-tune flowering time through a PHYB–FOF2–VOZ2 module that regulates FLC expression in response to shade [190,191].

### 4.3. Light Quantity/Photosynthesis Pathway

The quantity of light, including BL, can also influence plant flowering. Normally, increased light levels usually have a positive effect on flowering, despite varying responses among plant species [21]. The light quantity pathway is particularly crucial during early development, especially in herbaceous species with a distinct juvenile phase; it has been found in petunia that lower irradiances prolong the juvenile phase [21,192].

**Figure 5:**
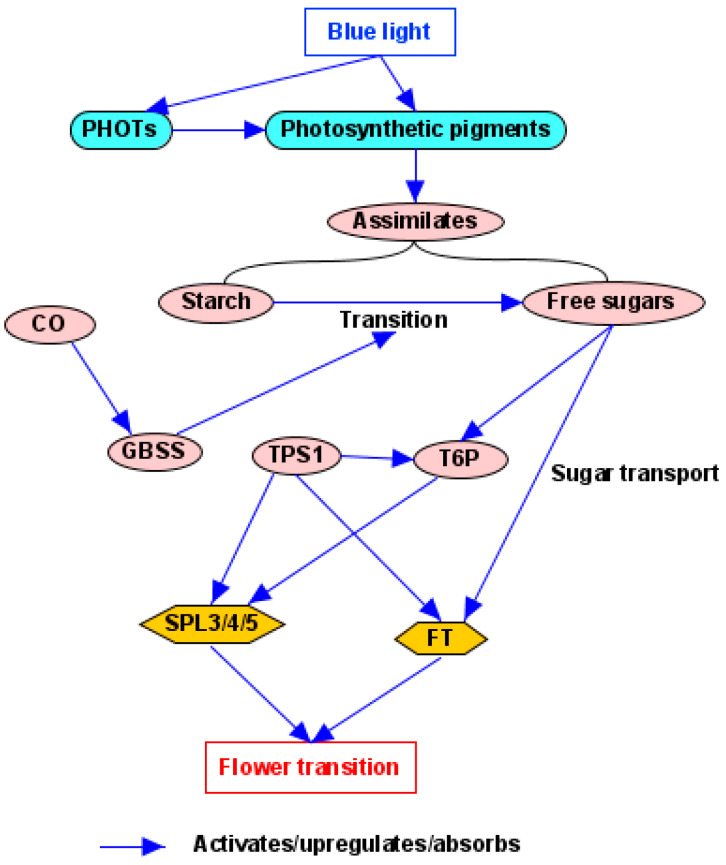
A simplified diagram of the light quantity pathway (or photosynthesis pathway) in blue-light-mediated floral transition. FT = FLOWERING LOCUS T, a florigen; SPL/3/4/5 = SQUAMOSA PROMOTER BINDING-LIKE 3/4/5, a group of positive floral integrator proteins. PHOTs = phototropins, blue light photoreceptors. CO = CONSTANS, a central component in the photoperiod pathway; T6P = trehalose-6-phosphate, a carbohydrate from sucrose; TPS1 = T6P synthase1; GBSS = GRANULE BOUND STARCH SYNTHASE. To be clear, only the key pathway components are presented in the diagram, and information about other pathway components can be found in the manuscript’s text. Also, the regulations at the transcriptional and translational levels are not distinguished in the figure for simplification, and the relevant information can be found in the text.

In general, flowering responses to light quantity are assumed to be linked to photosynthesis and the availability of assimilates, so the light quantity pathway is also called the photosynthesis pathway [21,193]. A strong irradiance dependence on flowering in *Brassica campestris* can be eliminated by supplying sucrose [21]. Because photosynthesis contributes to flowering regulated by the light quantity pathway, in this case, the photoreceptors are photosynthetic pigments [21,193] (Figure 5). As receptors for the regulation of flowering, photosynthetic pigments may sense a change in light quantity to affect photosynthesis and thus the availability of assimilates [105]. The contribution of the light quantity pathway to BL-mediated flowering results from the effects of BL on photosynthesis, which are associated with a very high absorbance of BL by photosynthetic pigments [63]. BLs also act on chloroplast movement within the plant cell to improve photosynthesis efficiency through its photoreceptor, PHOT [5,63].

For the light quantity pathway, assimilates act as part of a complex flowering signal [21]. The signals from assimilates may differ depending on the amount of light. Starch metabolism was differentially regulated during the floral transition in response to the amount of light, and the distribution of sugar and starch is linked to the floral transition [105,194,195]. As major assimilates, free sugars serve as important signaling molecules to regulate plant flowering [196]. Exposure to different light amounts changes the sugar content in *Ranunculus asiaticus*, showing a positive correlation between early flowering and higher accumulation of free sugars [197]. As a signal to initiate flowering, sugar type also varies across plant species [105]. For example, sucrose accumulation in the phloem increased during floral induction in *Sinapis alba* [198], but *Arabidopsis* has a higher demand for glucose and fructose than sucrose in the reproductive stage [199].

In addition to sucrose, glucose, and fructose, other carbohydrates may also play a role in the floral transition [105]. For example, trehalose-6-phosphate (T6P) accumulation is induced by sucrose, and T6P content is regulated in plants by T6P synthase (TPS) and T6P phosphatase (TPP) [200]. T6P signaling regulates flowering time in two different tissues; one is in the leaf where TPS1 is responsible for the induction of FT and TSF in response to photoperiod, and another one is in the SAM, where TPS1 and T6P signaling regulates the floral transition by controlling the transcription level of SPL3/4/5 via the age pathway, independent of the photoperiod pathway [105,201]. This indicates that, at least through T6P, the light quantity pathway can be integrated into other signal pathways.

Besides T6P, the light quantity pathway can potentially interact with the photoperiod pathway through light signal and/or pathway components. The amount of light is directly related to the photoperiod. The photoperiod can regulate starch metabolism differentially during the floral transition, and a disturbance in starch metabolism causes a change in flowering time [202,203]. CO may play a crucial role in the balance between free sugars and starch during floral transition by controlling the timing and the expression levels of GRANULE BOUND STARCH SYNTHASE (GBSS) [105]. Additionally, as an important photoreceptor in the photoperiod pathway, PHYA’s action is mediated partly and indirectly through photosynthesis, as flowering was delayed under low irradiance in *phyA* mutants but not at higher irradiances [21,193]. A recent study on chrysanthemum has also found that plant flowering responds to the intensity of prolonged photoperiod lighting (e.g., NI lighting) with BL, suggesting the co-regulation of photosynthetic carbon assimilation and differential photoreceptors in flowering [204].

Although assimilates themselves are part of a complex flowering signal, it is also possible that a sufficient mass flow of assimilates can contribute to the delivery of mobile flowering signals like FT [21]. In this case, besides sugar accumulation, carbohydrate transport is an important factor during the floral transition [105]. For example, chrysanthemum treated with exogenous sucrose showed a high induction of CmFTLs and FT homologs and flowered early under both LD and SD conditions [205]. Nevertheless, the precise molecular mechanisms through which light quantity, potentially through photosynthetic assimilation, modifies the length of the juvenile phase remain less clear.

## 5. Key Midstream Pathway Components

**Figure 6:**
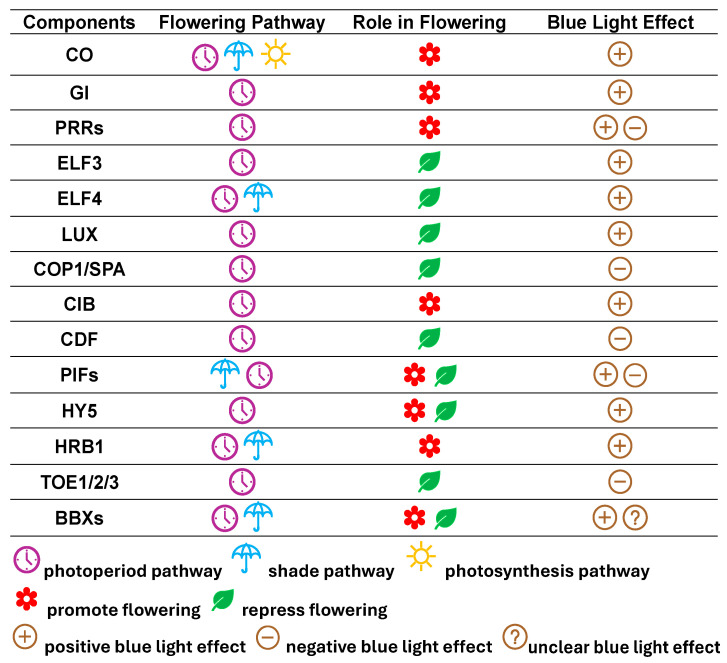
An illustration of the functions of key pathway components in blue-light-mediated flowering in terms of their involved flowering pathways, roles in flowering, and response to blue light. CO = CONSTANS; GI = GI-GANTEA; PRRs = PSUEDO RESPONSE REGULATORS; ELF3/4 = EARLY FLOWERING3/4; LUX = LUX ARRHYTHMO; COP1/SPA = CONSTITUTIVE PHOTOMORPHOGENIC 1/SUPPRESSOR OF PHYTOCHROME A; CIB = CRYPTOCHROME-INTERACTING basic helix–loop–helix; CDF = CYCLING DOF FACTOR; PIFs = PHYTOCHROME INTERACTING FACTORS; HY5 = ELONGATED HYPOCOTYL 5; HRB1 = Hypersensitive to red and blue protein; TOE1/2/3 = TARGET OF EAT1/2/3; and BBXs = B-box containing proteins. If both flowering promotion and repression are shown together, this indicates that the role of this pathway component depends on specific pathway or member. If both positive and negative effects of blue light are shown together, this indicates that the blue light effect may vary with light intensity or component member. For BBXs, although some members show positive responses to blue light, other members are unknown.

In the above three flowering pathways, after the reception of light signals by the photoreceptors, many midstream network components are involved in further transducing light signals to floral integrator proteins and eventually inducing flower initiation. The roles of some key midstream network components and their responses to BL are summarized as follows (Figure 6).

### 5.1. CONSTANS (CO)

CO is an important component in the photoperiodic flowering pathway, but the role of CO in photoperiodic flowering may vary with photoperiod, plant species, and light quality. In *Arabidopsis*, CO normally has a positive role in flowering induction in LD conditions by upregulating FT expression [156,157]. However, CO also has a negative role in flowering induction in SD conditions [206], where CO can inhibit flowering hypothetically through the flowering repressor TFL1, ensuring an alternative mechanism that prevents *Arabidopsis* from flowering in the wrong season [26]. In other plant species, proteins with a structure like that of CO (COL proteins) have been identified and studied in diverse crops, including ornamental plant, such as *Petunia hybrida* and *Pharbitis nil*, and economically important crops, including rice, barley, wheat, maize, sorghum, cotton, hemp (*Cannabis sativa* L.), tomato, grapevine (*Vitis vinifera*), and strawberry [157]. For example, in rice, there are two important COL transcription factors: Heading date1 (Hd1), an ortholog of the *Arabidopsis* CO, and Early heading date1 (Ehd1), which is unique in rice [119]. Ehd1 always acts as an inducer of florigen genes (*Hd3a* in SD conditions or *RFT1* in LD conditions), and its expression is upregulated by BL in the morning; however, Hd1 acts as a repressor in noninductive LD [157]. In strawberries, although both blue and FR light treatments trigger flowering through the FvFT1 cascade, BL operates through FvFT1 and is partially independent of FvCO, while FR light operates completely independently of FvCO [127].

BL, like FR light, enhances the stability of CO proteins, but red light promotes the degradation of CO [156,158]. For example, in *Hippeastrum hybridum* under RB-LED lighting with 10% or 90% B, RB-LED with 90% B compared with white LED advanced flowering time and extended the duration of the flowering period, which was associated with greater expression of the *HpCOL* gene [207]. The stabilization of CO by BL is a balanced result of the contribution of several BL receptors. FKF1, identified as a CDF-destabilizing photoreceptor, interacts directly with CO proteins to enhance their stability [156]. FKF1 also prevents COP1 homodimer formation, reducing COP1-dependent CO degradation [208]. Additionally, FKF1 competes with AP2-like proteins (TOE1, TOE2, TOE3), known FT repressors, for CO protein stabilization [156,209]. The FKF1′s role in CDF degradation and CO protein stabilization is crucial for photoperiodic sensitivity [210]. Similarly to FKF1, BL photoreceptors CRY1 and CRY2 can also enhance the stability of CO proteins by physically impairing TOE1, TOE2, and the COP1–SPA complex [156]. However, ZTL leads to CO degradation [173], although the mechanism remains unclear.

### 5.2. Circadian Clock

Photoreceptors influence flowering time indirectly by modulating the circadian clock [193], a gene and protein network producing 24 h oscillations synchronized to environmental cycles [211]. This internal timing system allows plants to anticipate and adapt to daily changes in the environment [212]. The circadian clock comprises complicated components, and BL can regulate the clockwork and its components to regulate plant flowering.

#### 5.2.1. Clock Components

**Figure 7:**
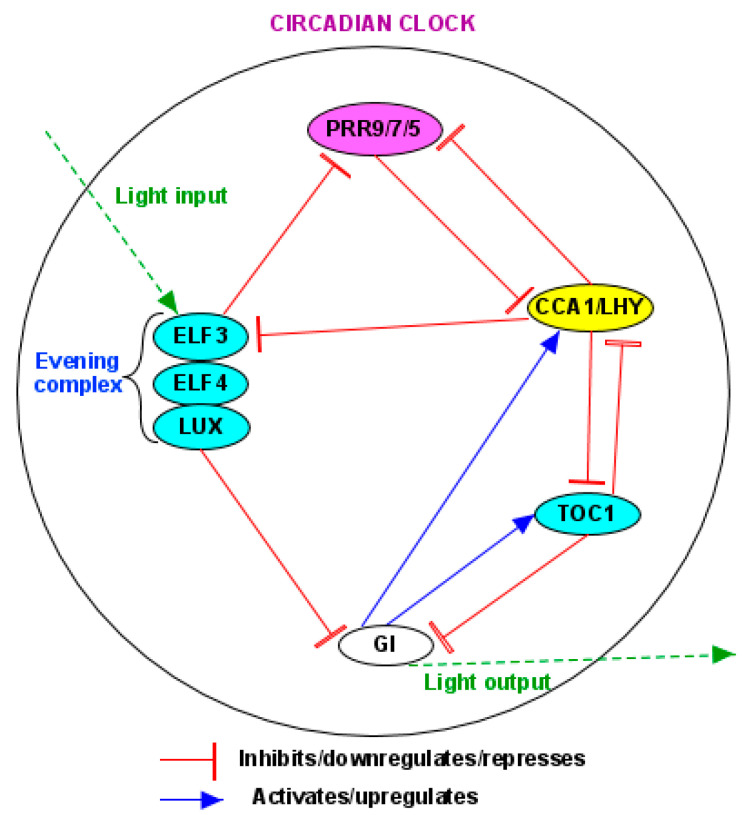
A simplified diagram of the main components of the circadian clock in *Arabidopsis thaliana*. The figure is adapted and modified from the literature [170]. CCA1/LHY = CIRCADIAN CLOCK ASSOCIATED 1/LATE ELONGATED HYPOCOTYL; PRR9/7/5 = PSUEDO RESPONSE REGULATOR9/7/5; TOC1 = Timing of CAB2 Expression 1; LUX = LUX ARRHYTHMO; ELF3/4 = EARLY FLOWERING 3/4; GI = GIGANTEA. Among these clock components, ELF3 and GI are important clock input and output members, respectively. In the diagram, the yellow components are expressed in the morning, purple components are expressed midday, and blue components are expressed in the evening.

The plant circadian clock is composed of three main parts: (1) input pathways that perceive and transmit environmental signals, (2) a central oscillator that generates 24 h rhythms, and (3) output pathways that regulate physiological processes [158]. In *Arabidopsis*, the identified core clock proteins include at least morning-expressed proteins CIRCADIAN CLOCK ASSOCIATED 1 (CCA1) and LATE ELONGATED HYPOCOTYL (LHY); midday regulators PSEUDORESPONSE REGULATOR 9 (PRR9), PRR7, and *PRR5*; and evening-expressed components EARLY FLOWERING 3 (ELF3), ELF4, and LUX ARRHYTHMO (LUX), which are called the evening complex, and Timing of CAB2 Expression 1 (TOC1) [213]. These components, together with GI, form an interlocked feedback loop to control circadian rhythms (Figure 7). The function of these core circadian components is largely conserved among *Arabidopsis* and crops like rice, maize, and duckweed (*Lemna gibba*) [119]. Among these clock components, CCA1, LHY, ELF3, ELF4, and LUX act as flowering repressors, but PRR9, PRR7, PRR5, and TOC1 positively regulate flowering in *Arabidopsis* [163].

#### 5.2.2. Regulation of Clockwork by BL

Blue light entrains the circadian oscillator by adjusting its pace and phase. In *Arabidopsis*, the circadian period shortens under BL compared to darkness (where the plant has a circadian period of 30 to 36 h), with increased intensity accelerating the rhythm (or shortening the circadian period [72,170,214].

Photoreceptors CRY1, CRY2, and PHYA serve as primary BL sensors for the clock’s entrainment [82]. Normally, PHYA is most important in low-intensity blue light, CRY2 in low-intensity BL, and CRY1 in high- or low-intensity BL [215]. For example, PHYA is necessary to re-entrain the oscillator in response to a BL pulse at the end of the night, which shifts the phase of the clock [82]. Additionally, some other BL photoreceptors, such as the ZTL family, have also been shown to regulate clock components [193]. The mechanism linking BL perception to the core of the circadian clock is still not fully understood. In *Arabidopsis*, CRYs have been revealed to affect the circadian period through photo-signaling hub factors, PIFs, COP1, or other transcription factors [214]. For example, CRY2 binding to several core clock gene promoters for fine-tuning the oscillator could be modulated by PIF due to a proven interaction between CRYs and PIFs [176]. COP1, acting at the crossroads of multiple light wavelengths, also conveys BL information to the circadian clock [82]. Also, BL can repress the transcription of *COLD REGULATED GENE 27 (COR27)* and *COR28* but stabilize COR27 and COR28 proteins, which bind to the promoters of *PRR5* and *TOC1* and repress their transcription [216]. Additionally, HY5, induced by BL, binds directly to clock gene promoters, including *CCA1*, *PRR9*, and *LUX*, integrating BL perception with clock function [82,189,211]

#### 5.2.3. Responses of Key Clock Components to BL

(1) BL and GI

GI serves as a core component that transmits BL signals for circadian rhythm resetting while overseeing floral initiation [162]. In *Arabidopsis*, under BL conditions, GI can bind to ZTL and establish a GI–ZTL protein complex to stabilize ZTL expression [217]. In the BL signaling system, GI binding to ZTL as well as LKP2 to orchestrate the degradation of TOC1 and PRR5, contributing to correct oscillation of the plant’s circadian clock [93,162,218]. BL also induces the formation of an FKF1–GI protein complex to promote CO and FT expression and flowering in LD conditions [219,220], as mentioned in Section 4.1. In rice, OsGI plays a critical gatekeeper role in BL induction of Ehd1, a CO-like protein, resulting in early flowering under SD conditions [131,221]. In chrysanthemums, CsGI can control photoperiodic flowering by shaping the gate for light induction of CsAFT, an anti-florigen [121], but the specific response of CsGI to BL is unknown.

(2) BL and PRRs

PRR9 and PRR7 act redundantly not only as a crucial circadian output hub to control photoperiodic flowering time but also participate in the light input pathway [214,222]. Specifically, PRR9 mainly mediates BL signal input, while PRR7 acts in red light input [222]. Recently, it has been found that photoreceptor CRY2 mediates BL input to the circadian clock by directly interacting with PRR9 in a BL-dependent manner [214]. Given the labile nature of CRY2 in strong BL, BL triggers CRY2 turnover in proportion to its intensity, which accordingly releases PRR9 to fine-tune circadian speed [214].

(3) BL and evening complex (EC)

ELF3 is involved in BL-mediated circadian regulation [168]. Under continuous blue or red light conditions, overexpression of ELF3 led to a significant extension of the circadian period across a wide range of fluence rates, resembling the fluence response curves observed in plants lacking functional CRY1 and CRY2 [223]. ELF3 is also part of the BL signaling pathway through its interaction with COP1, and some, but not all, inputs of PHYs to the clock depend on ELF3 [170]. So far, no BL receptors have been identified that directly interact with ELF3 [224]. However, BL can promote the localization of ELF3 to foci independent of ELF4 [225]. ELF4 also negatively regulates flowering by affecting the biological activity of the GI. In petunia, two ELF4-like genes, *PhELF4-1* and *PhELF4-2*, are under photoperiodic control involving the circadian clock and implicated in signal transduction from one or more BL photoreceptors [10]. Both genes exhibit a rhythmic expression pattern, with higher expression under blue LED light compared to red LED light, peaking 12 h into a 14 h photoperiod. *PhELF4-1* shows higher amplitude and higher peak expression than *PhELF4-2*. *PhELF4-1* maintains its expression rhythm for two days under continuous blue LED light, indicating a circadian rhythm, and shifts phases after transfer to short days with an 8 h photoperiod [10].

LUX acts as a flowering repressor [158,163]. The expression of LUX is activated by BL, in addition to cold temperatures and red light [225]. The activation of LUX expression by blue and red light occurs through HY5, and, in this process, BL has a major effect, while red light has a minor effect [225].

### 5.3. Other Key Transcription Factors/Regulators

#### 5.3.1. CONSTITUTIVE PHOTOMORPHOGENIC 1/SUPPRESSOR OF PHYTOCHROME A (COP1/SPA)

As a downstream pathway component after photoreceptors, the COP1/SPA complex is a master repressor of light signaling by ubiquitinating and subsequently degrading many light-regulated transcription factors (e.g., CO, PIFs, and HY5) in darkness [95]. Under light conditions, photoreceptors, such as CRYs and PHYs, suppress the activity of the COP1/SPA complex [172]. For the COP1/SPA complex, SPA proteins are required for the light-induced nuclear exclusion of COP1 and thereby contribute to the light-induced inhibition of COP1 [226]; however, blue or red light leads to dissociation of the COP1–SPA1 interaction, likely a very important mechanism of light-induced inhibition of COP1/SPA activity [172]. In *Arabidopsis* under BL, COP1/SPA activity is inhibited by CRY1 and CRY2, FKF1, and PHYA [95]. In addition to FKF1, the other members of the ZEITLUPE family, such as ZTL and LKP2, also interact with COP1 [95,208]. This leads to the stabilization of the CO protein, followed by early flowering under LD conditions [208]. Following this mechanism, these BL photoreceptors may act in parallel or together in promoting flowering via CO [95]. Moreover, COP1/SPA facilitates the light-induced degradation of PIFs by associating with PHYs [172]. In *Arabidopsis*, COP1/SPA ubiquitinating HY5 is suppressed by BL through CRYs; however, in *Marchantia polymorpha*, MpCRY is not an inhibitor of this process under BL, although COP1/SPA ubiquitinating HY5 is conserved in this species [227].

#### 5.3.2. CRYPTOCHROME-INTERACTING Basic Helix–Loop–Helix (CIB) Proteins

The CIBs, belonging to basic helix–loop–helix (bHLH) proteins, are important downstream components after photoreceptors in the photoperiodic flowering pathway, and BL mediates CIB proteins through its photoreceptors to regulate flowering. CRY2 mediates the BL stimulation of CIB1 activation, which promotes the transcription of the *FT* gene, and three CIB1-like proteins (CIB2/4/5) also interact with photoactivated CRY2 and bind to the *FT* promoter [28]. Furthermore, CIB proteins directly interact with CO to promote floral initiation, suggesting that CRY2 might influence the CIB–CO interaction and *FT* transcription [228]. In the dark or under red light, CIB proteins (CIB1/4/5) are degraded by the 26S proteasome; however, BL suppresses their degradation through ZTL and LKP2, rather than through CRYs [28].

#### 5.3.3. CYCLING DOF FACTOR (CDF)

As an important component of the photoperiod pathway, the CDF family members (especially CDF1) act as floral repressors under LD conditions by inhibiting CO expression in the first part of the light period [137]. In the LD afternoon, a protein complex formed by GI and FKF1 is required to degrade the CDF proteins, releasing the repression of CO and FT transcription [159]. In *Arabidopsis*, FKF1 and GI form a complex in a BL-dependent manner, which mediates the degradation of the CDF1 protein [219]. The function of FKF1 depends on the interaction between GI and CDF1, and GI also interacts with ZTL and LKP2 and synergistically degrades CDF2 protein [160,173,174].

#### 5.3.4. PHYTOCHROME INTERACTING FACTORS (PIFs)

The PIFs, belonging to bHLHs, can also mediate flowering by binding their genes to floral integrator proteins as well as other flowering regulators, such as AP1, SVP, LFY, AGAMOUS (AG), and SEP3 (SEPALLATA3) in *Arabidopsis* [229]. In addition to *Arabidopsis*, in rice, *ospil13*, one of the putative PIF4 homologs, mutants were reported to head earlier compared to the wild type, indicating that OsPIL13 might regulate floral development in rice [230,231].

The PIFs are involved in multiple flowering pathways, including the shade pathway and the photoperiod pathway, as mentioned before. PIF7 functions as the major regulator alongside PIF1/3/4/5 in orchestrating shade avoidance syndrome (SAS) and thus indirectly mediating flowering [232]. Also, PIFs interact with circadian clock components like TOC1 to modulate the timing of gene expression, which ensures that flowering time regulators are expressed in coordination with the circadian rhythm [229]. PIF7, together with CO, plays an additive role in the promotion of flowering under shade conditions through the induction of FT expression [26,177]. Additionally, the PIFs are also involved in other signaling pathways to regulate flowering. For example, when there is an increase in the ambient temperature, PIF4 is stabilized, and it directly binds to the promoter of *FT* and induces its expression, indicating that FT can also induce flowering in a temperature-dependent manner via PIF4 [230]. PIF1 suppresses the floral transition in *Arabidopsis* under LD conditions by interacting with gibberellins (GAs) and floral integrators, such as *FT*, *SOC1*, and *LFY* [233]. Furthermore, under shade conditions, GA-mediated degradation of DELLA relieves their inhibition of PIF4, which in turn activates *FT* transcription to promote flowering [182].

BL-activated PHYA induces the degradation of PIF1 [234], a suppressor of floral transition, and thus possibly promotes flowering. However, no evidence supports BL-triggered, CRY-induced degradation of PIF1 [235]. Also, CRYs directly interact with PIFs, and this interaction modulates the activity of PIFs, thereby affecting flowering [182]. It has been shown that PIF4 and PIF5 are CRY-interacting proteins and that PIF4, PIF5, and CRY2 bind to common chromatin regions of target genes [176].

#### 5.3.5. ELONGATED HYPOCOTYL 5 (HY5)

The HY5 is a signaling hub component acting downstream of several photoreceptors [211]. HY5 regulates the circadian clock by binding to specific promoter elements of the majority of clock genes [236]. Also, HY5 interacts with histone deacetylase HDA9 to epigenetically regulate flowering time in *Arabidopsis* by repressing the expression of *PIF4* and *COL5* [237,238].

Under BL, activated CRYs interact with and inhibit COP1, which targets HY5 for degradation, which leads to the stabilization and accumulation of HY5 [236]. BL is more efficient than red light in inducing the accumulation of HY5 at transcriptional and post-transcriptional levels, and HY5-mediated signaling to the clock is most pronounced in BL [211]. Therefore, BL could efficiently modulate the pace of the circadian clock by governing the abundance of HY5 [211].

#### 5.3.6. Hypersensitive to Red and Blue Protein (HRB1)

The HRB1 is a downstream component after photoreceptors to regulate flowering in at least the photoperiod pathway and the shade pathway. The *hrb1* mutant flowers late under both LD and SD conditions, whereas *HRB1* overexpression leads to early flowering under SD conditions due to altered *FT* expression [158]. HRB1 regulates CRY-mediated BL responses as well as PHYB-mediated red light responses and is localized to the nucleus [158]. HRB1 may regulate *FT* expression and flowering downstream of CRY2 in LD conditions and modulate the PHYB-mediated shade pathway to regulate flowering in both LD and SD conditions [239]. HRB1 is essential for the proper expression of *PIF4* under red light and BL, suggesting that HRB1 and PIF4 may be crucial intersection points for red light and BL signaling pathways [158]. Additionally, *HRB1* expression is regulated by the circadian clock, similarly to other flowering genes [158].

#### 5.3.7. TARGET OF EAT1/2/3 (TOE1/2/3)

At least involved in the photoperiod pathway, the APETALA2 (AP2)-like proteins, TOE1/2/3, are well-known FT repressors and compete with FKF1 for CO, resulting in CO destabilization due to a lack of access to FKF1 proteins [209]. Similarly to FKF1, CRY1 and CRY2 physically impair TOE1 and TOE2 under BL [156,240].

#### 5.3.8. B-Box Containing Proteins (BBXs)

The BBXs are transcription factors essential in plant flowering [241]. The first BBX protein characterized and identified in *Arabidopsis* was CO/BBX1 [242]. The following BBXs mean proteins other than CO/BBX1.

The BBXs can not only inhibit photoperiod-mediated flowering but also regulate the shade avoidance response (SAR) to control flowering. For example, BBX10, BBX14, BBX15, BBX16, BBX17, BBX19, BBX30, and BBX31 interact with CO to inhibit its expression, while BBX32 forms heterodimers with BBX4 to bind and repress the *FT* promoter, and BBX7 inhibits the expression of both *CO* and *FT* [241,243]. Also, BBX21 upregulates genes involved in PHYB, BR, and auxin signaling to inhibit SAR, while BBX24 and BBX25 promote SAR in a COP1-dependent manner and BBX24 interacts with DELLAs and JAZ3 to modulate PIF4 activity and DELLA suppression, promoting SAR [241].

BL can affect levels of BBXs in plants. In *Arabidopsis*, BBX17, as a flowering repressor under LD conditions, is degraded in the dark but stabilized under BL as well as white, red, or FR light [242]. In strawberries, among the identified BBX proteins, the downregulated FaBBX29 plays an important role in the flowering promoted by sole-source lighting with blue vs. white light [160]. In *Arabidopsis*, CRY2, activated by BL, competes with HY5 to interact with COP1, thereby stabilizing HY5 protein, which directly targets the promoters of *BBX7* and *BBX8* to positively regulate their expression [244]. The CRY2-COP1-HY5-BBX7/8 module regulates BL-dependent cold acclimation, but whether the module can also mediate flowering is unknown.

## 6. Concluding Remarks and Future Perspectives

Floral transition is a crucial physiological process in plants, and regulation of this process directly affects the productivity of many crops [1,245]. Although significant progress has been made in understanding BL-mediated flowering, further mechanistic research is essential to deepen our scientific knowledge in this area. Much of our current understanding comes from studies on the model plant *Arabidopsis*, which, while valuable, has no direct agricultural importance and offers limited insight for SD or day-neutral crops. In contrast, only fragmental information is available on horticultural crops, as well as field/agronomic crops, which can be found in different sections of this manuscript and has also been summarized in Table 1. This limits the application of BL to flowering regulation, especially in horticultural crops, which are the most common crops produced in a controlled environment.

Emerging evidence highlights substantial variation in BL responses between model and crop species. For instance, a recent study has revealed that tomato, a widely cultivated crop, is relatively insensitive to BL in terms of flowering, unlike *Arabidopsis*, which shows sensitive responses [250]. This discrepancy underscores the growing need to investigate BL-mediated flowering directly in agriculturally important species. Despite beginning, increased research interests have been focused on exploring mechanisms underlying BL-regulated floral transition in horticultural crops, especially chrysanthemums and strawberries.

Chrysanthemum, a quantitative SD plant, typically fails to flower under LD conditions. However, extended photoperiod lighting using blue LEDs did not inhibit floral transition and even promoted stem elongation, which is desirable for cut flower production [8]. Research from Korea has shown that BL treatment activates specific photoreceptors, enhances sugar accumulation, and increases expression of FT, all contributing to flowering responses [16,114,204,246]. Nevertheless, the effects of BL in chrysanthemums are highly dependent on environmental conditions and vary among different SD plant species [8,247,248], indicating a need for deeper exploration of the underlying regulatory mechanisms.

In strawberries, BL has also been identified as a crucial signal in flowering regulation [127,128,142,146,160]. For example, sole-source blue lighting has been shown to promote flowering more effectively than white light [160]. Under BL, genes involved in light signaling (*PHYB*, *PIFs*, and *HY5*) and the circadian clock (*FKF1*, *CCA1*, *LHY*, and *CO*) are differentially expressed. Also, the BBX family protein, FaBBX29, was found to play an important role in flowering regulation under BL. Additionally, contrasting results about the expression of FaTFL1 have been reported in short-day varieties [142,146]. This suggests that a complex signaling network may be involved in strawberry’s flowering response to BL.

Overall, BL-mediated flowering involves multiple photoreceptors and intricate signaling pathways. This complexity likely contributes to the diverse flowering responses observed under BL treatments in controlled environments. Therefore, applying BL to regulate flowering should consider many factors, such as species-specific and environmental differences. Continued in-depth mechanistic studies, particularly in horticultural crops, are essential to fully use BL as a practical tool in crop production.

## Figures and Tables

**Table 1:** Summarized mechanistic studies related to blue-light-mediated flowering in horticultural crops and field/agronomic crops in this review.

Common Name	Scientific Name	Main Results	Reference(s)
1. Horticultural crops			
Chrysanthemum	*Chrysanthemum morifolium*	Blue light (BL) did not inhibit floral transition under long day (LD), despite increased expression of both *PHYA* and *PHYB*.	[16]
		Shifts in the sequence of blue and other LEDs for night interruption (NI) lighting caused varying flowering responses associated with different expressions of related genes, such as *PHYA*, *CRY1*, *PHYB*, *AFT*, and *FTL*.	[114]
		At least two distinct phytochrome responses were involved in the flowering response; daytime light quality affects the light quality required for effective NI lighting due to different effects on FLOWERING LOCUS T (CmFTL3).	[111]
		An anti-florigen gene, *AFT*, was identified to contribute to the phytochrome (PHY)-mediated response to light and to determine the obligate photoperiodic flowering response.	[141]
		Flowering-promotion responses by long day BL treatments resulted from balanced expression of a series of genes, such as *CmFTL3*, *APETALA1 (CDM111)*, *FRUITFULL (CmAFL1)*, *LEAFY (CmFL)*, *CmPHYA*, *CmCRY1*, *CmAFT*, and *CmPHYB*. Also, the youngest leaf showed greater sensitivity to BL.	[148]
		BL-promoted flowering might be due to the co-regulation of photosynthetic carbon assimilation and differential photoreceptors in flowering.	[204]
		Increased photosynthesis, carbohydrate accumulation, and antioxidant production contributed to BL-promoted flowering.	[246]
		Exogenous sucrose induced a high expression of CmFTLs, and it flowered early regardless of the photoperiod.	[205]
		CsGI controlled photoperiodic flowering by gating light-induced CsAFT.	[121]
		A high daytime phytochrome photoequilibrium prevented plants from perceiving subsequent BL as a long day.	[247]
		Plant flowering under long-day BL treatment was not observed in all short-day (SD) plants.	[248]
Baby’s Breath	*Gypsophila paniculata*	BL did not induce FT and SOC1 expression and had weaker flowering promotion than far-red (FR) light.	[14]
		Genotype variation in the flowering response to blue light was associated with GpFT and GpSOC1, rather than GpFKF1 and GpGI.	[11]
Kalanchoe	*Kalanchoe blossfeldiana*	BL was not perceived as a photoperiod signal to regulate flowering, which was mainly controlled by red light.	[112]
		The BL signal at the end of day increased flower bud formation regardless of the photoperiod, which was associated with higher expression of flowering promoter genes (*KfPHYA*, *KfCRY1*, *KfFT*, and *KfFPF-1*) and lower expression of the flowering suppressor gene (*KfPHYB*).	[147]
Lisianthus	*Eustoma grandiflorum*	Prolonged photoperiod lighting with BL promoted flowering under SD, associated with increased expression of EgFTL and EgSOC1L, but there was weaker promotion compared to FR light.	[135]
Marigold	*Tagetes erecta*	Twenty-four-hour sole-source lighting with pure BL promoted flowering compared with red light; however, impure BL containing a low level of red light failed to induce flowering, and adding a low level of far-red light restored the flowering-promoting effect.	[6,7]
Petunia	*Petunia × hybrida*	BL influenced *PehFT* expression but not the main gene promoting flowering.	[132]
		Blue vs. red LED light increased the expression of *FBP28*, a *SOC1*-like gene, which transmitted the BL signal from the FT protein to induce flowering.	[10,249]
		Two *ELF4*-like genes, *PhELF4-1* and *PhELF4-2*, were identified to act in signal transduction from one or more BL photoreceptors.	[10]
		Lower irradiances prolonged the juvenile phase.	[192]
		Twenty-four-hour sole-source lighting with pure BL promoted flowering compared with red light; however, impure BL containing a low level of red light failed to induce flowering, and adding a low level of far-red light restored the flowering-promoting effect.	[6,7]
*Phalaenopsis* orchid	*Phalaenopsis* spp.	Flowering response depended on active PHY levels under supplemental lighting.	[113]
Strawberry	*Fragaria × ananassa*	Sole-source lighting with blue vs. white LEDs promoted flowering associated with altered expression of PHYB, PIFs, HY5, FKF1, CCA1, LHY, and CO. The downregulated FaBBX29, an identified BBX protein, played an important role in BL-promoted flowering.	[160]
		Both blue and FR light promoted flowering in day-neutral accessions through FvFT1, but BL acted partially independent of FvCO, and FR light was completely independent of FvCO. Also, BL induced the expression of FvFT1 exclusively in veins of older leaves.	[127]
		Blue LED light increased the number of flower clusters and final yield in an everbearing variety.	[128]
		End-of-day lighting with BL or FR light could induce a higher expression of FvTFL1, the repressor of floral induction, in an SD variety.	[142]
		Blue LED light promoted the flowering of an SD variety in LD conditions, associated with increased expression of FaFT1 and decreased expression of FaTFL1, as well as enhanced photosynthesis and carbohydrate production.	[146]
Sweet pepper	*Capsicum annuum*	Sole-source lighting with BL induced the expression of CaFT1 and CaFT2 compared to white light.	[129]
Tomato	*Solanum lycopersicum*	BL sensitivity differs from *Arabidopsis*; indifferent flowering response found.	[250]
		Both CRY2 and CRY1a function to repress tomato flowering: knockout of *CRY2* or *CRY1a* does not affect flowering time, but the simultaneous knockout of both *CRY1a* and *CRY2* promotes flowering.	[46,52]
		This species has at least three *CRY* genes, *CRY1a*, *CRY1b*, and *CRY2*.	[31,32]
		Under LD conditions, *CRY2* overexpression in this species delayed flowering.	[48]
2. Field/agronomic crops			
Barley	*Hordeum vulgare*	CRY1a and CRY2a were identified, and CRY1a was a major regulator of photoperiodic flowering.	[32]
Maize	*Zea mays*	PHYB2 and, to a lesser extent, PHYB1 mediated photoperiodic flowering, and the sub-functionalization might contribute to flowering variation among varieties.	[59]
Onion	*Allium cepa*	Overexpression of *AcCRY1* accelerated flowering; BL promoted cytoplasmic localization.	[36]
Rice	*Oryza sativa*	Knockdown of *CRY2*, but not *CRY1*, delayed flowering both in long- and short-day conditions.	[46]
		Overexpression of *CRY2* in plants with a photoperiod-insensitive genetic background did not affect flowering time.	[47]
		PHYC functioned as a flowering repressor under noninductive photoperiods.	[76]
		FKF1 had a similar role in photoperiod-mediated flowering relative to *Arabidopsis* and promoted flowering independent of the photoperiod.	[91]
		OsHAL3 was identified as a new BL sensor, which was structurally inactivated by light, especially BL.	[99]
		OsHAL3 was a positive regulator of flowering by directly binding to the promoter of *Hd3a* and forming a complex with Hd1 under SD conditions.	[100]
		Hd3a and RFT1 are essential for promoting rice flowering under SD conditions, while RFT1 functions as a floral activator under LD conditions.	[119]
		Both Hd3a and RFT1 are expressed in leaves and move to the SAM, where they enhance the expression of floral meristem identity genes and trigger flowering.	[119,124,125]
		NI lighting with BL suppressed Hd3a expression and delayed flowering.	[130]
		RCN (a TFL1 homolog) inhibited flowering by competing with Hd3a (a FT homolog) for 14-3-3 binding to form a florigen repression complex.	[118,144]
		There are two important COL transcription factors. These are Heading date1 (Hd1), an ortholog of the *Arabidopsis* CO, and Early heading date1 (Ehd1) which is unique in rice.	[119]
		*Ehd1* always acts as an inducer of florigen genes (*Hd3a* in SD conditions or *RFT1* in LD conditions), and its expression is upregulated by BL in the morning; however, *Hd1* acts as a repressor in noninductive LD.	[157]
		OsGI plays a critical gatekeeper role in BL induction of Ehd1, a CO-like protein, resulting in early flowering under SD conditions.	[131,221]
		OsPIL13 might regulate floral development; *ospil13*, one of the putative *PIF4* homologs, mutants headed earlier compared to the wild type.	[230,231]
Sorghum	*Sorghum bicolor*	CRY2 is a major regulator of photoperiodic flowering; CRY1b can rescue the late-flowering phenotype in *Arabidopsis cry1/cry2* double mutant.	[46,51]
Soybean	*Glycine max*	CRY1a rather than CRY2a is a major regulator of photoperiodic flowering.	[46,50]
		The regulation of photoperiodic flowering through PHYA-LUXE1-FT is different from the PHYB-CO-FT flowering pathway in many nonlegume plants.	[150,152]
Wheat	*Triticum aestivum*	PHYC promoted flowering under inductive photoperiods.	[75]

## Data Availability

Data are contained within the article.
